# Mathematical algorithm–based identification of the functional components and mechanisms in depression treatment: An example of Danggui-Shaoyao-San

**DOI:** 10.3389/fcell.2022.937621

**Published:** 2022-08-22

**Authors:** Wenxia Gong, Kexin Wang, Xueyuan Wang, Yupeng Chen, Xuemei Qin, Aiping Lu, Daogang Guan

**Affiliations:** ^1^ Modern Research Center for Traditional Chinese Medicine of Shanxi University, Taiyuan, Shanxi, China; ^2^ Key Laboratory of Chemical Biology and Molecular Engineering of Ministry of Education, Taiyuan, Shanxi, China; ^3^ Key Laboratory of Effective Substances Research and Utilization in TCM of Shanxi Province, Taiyuan, Shanxi, China; ^4^ National Key Clinical Specialty/Engineering Technology Research Center of Education Ministry of China, Guangdong Provincial Key Laboratory on Brain Function Repair and Regeneration, Department of Neurosurgery, Neurosurgery Institute, Guangzhou, China; ^5^ Institute of Integrated Bioinformedicine and Translational Science, Hong Kong Baptist University, Hong Kong, China; ^6^ Department of Biochemistry and Molecular Biology, School of Basic Medical Sciences, Southern Medical University, Guangzhou, China; ^7^ Guangdong Key Laboratory of Single Cell Technology and Application, Southern Medical University, Guangzhou, China

**Keywords:** mathematical algorithm, functional components, mechanism, Danggui-Shaoyao-San, depression, core function motifs

## Abstract

Depression, a complex epidemiological mental disorder, affects around 350 million people worldwide. Despite the availability of antidepressants based on monoamine hypothesis of depression, most patients suffer side effects from these drugs, including psychomotor impairment and dependence liability. Traditional Chinese medicine (TCM) is receiving more and more attention due to the advantages of high therapeutic performance and few side effects in depression treatment. However, complex multicomponents and multi-targets in TCM hinder our ability to identify the functional components and molecular mechanisms of its efficacy. In this study, we designed a novel strategy to capture the functional components and mechanisms of TCM based on a mathematical algorithm. To establish proof of principle, the TCM formula Danggui-Shaoyao-San (DSS), which possesses remarkable antidepressant effect but its functional components and mechanisms are unclear, is used as an example. According to the network motif detection algorithm, key core function motifs (CIM) of DSS in treating depression were captured, followed by a functional analysis and verification. The results demonstrated that 198 pathways were enriched by the target genes of the CIM, and 179 coincided with the enriched pathways of pathogenic genes, accounting for 90.40% of the gene enrichment pathway of the C-T network. Then the functional components group (FCG) comprising 40 components was traced from CIM based on the target coverage accumulation algorithm, after which the pathways enriched by the target genes of FCG were selected to elucidate the potential mechanisms of DSS in treating depression. Finally, the pivotal components in FCG of DSS and the related pathways were selected for experimental validation *in vitro and in vivo*. Our results indicated good accuracy of the proposed mathematical algorithm in sifting the FCG from the TCM formula, which provided a methodological reference for discovering functional components and interpreting molecular mechanisms of the TCM formula in treating complex diseases.

## Introduction

Depression, a complex epidemiological mental disorder, stands at the 9th position behind prolific killers such as heart disease, stroke, and HIV, when ranked based on disability and death (Smith, 2014). A latest survey by the World Health Organization shows that more than 350 million people worldwide are suffering from depression (World Health Organization, 2017; Liu et al., 2021). Depression is often accompanied by a depressed mood, cognitive impairment, anhedonia, disturbed sleep, and low energy (Nabavi et al., 2017), which not only causes enormous consequences on the health and life quality of patients but also directly results in death by suicide (Wang et al., 2019). Consequently, depression has become one of the most prominent public health problems. Many synthesized antidepressants have played important roles in the therapy of depression, such as monoamine oxidase inhibitors (MAOIs), tricyclic antidepressants (TCAs), and selective serotonin re-uptake inhibitors (SSRIs). However, these antidepressant drugs have several side effects, including psychomotor impairment and dependence liability. In these circumstances, traditional Chinese medicine (TCM) is receiving more and more attention due to the advantages of high therapeutic performance and few side effects in depression treatment (Li et al., 2020). Therefore, it is of great significance for the treatment of depression to excavate the functional components and analyze their potential mechanisms from TCM.

Danggui-Shaoyao-San (DSS), also called Toki-shakuyaku-san (TJ-23) or Dangguijakyak-san (DJS), is a widely used formula of traditional Chinese medicine derived from “Jin Kui Yao Lue” in the Eastern Han Dynasty of China (Fu et al., 2015). DSS is composed of *Angelica sinensis* (Oliv.) Diels (Danggui), *Poria cocos* (Schw.) Wolf (Fuling), *Alisma*×*bjoerkqvistii* Tzvelev (Zexie), *Paeonia lactiflora* Pall (Baishao), *Atractylis lancea var. chinensis* (Bunge) Kitam (Baizhu), and *Rhizoma Ligustici* Chuanxiong (Chuanxiong) at a proportion of 3:4:8:16:4:3 (Bi et al., 2021). It has been used throughout Asia for thousands of years on gynecological, cardiovascular, neurological, and mental diseases due to multiple pharmacological activities such as improving hemorrheology, inhibiting platelet aggregation, enhancing cognition, and affecting immune and neuroendocrine functions (Ahn et al., 2019; Seo et al., 2020; Luo et al., 2021; Shi et al., 2021). Recently, the pharmacological activity of this formula in depression has attracted attention. The TCM theory holds that depression is attributed to an imbalance or blockage in one or more of the internal organs such as liver stagnation and spleen deficiency (Liu et al., 2021). DSS is one of the representative formulae to harmonize the liver and the spleen (Xu et al., 2011). The clinical and pharmacological studies showed that DSS could relieve depressive symptoms in postmenopausal women (Koike et al., 2004; Terauchi et al., 2014) and experimental animal (Xu et al., 2011; Li et al., 2022). Terauchi et al. (2014) demonstrated that DSS was effective in treating the symptoms of headaches and concomitant depression in women. Xu et al. (2011) suggested that DSS exerted an antidepressant effect in the forced swimming test (FST) and chronic unpredictable mild stress (CUMS) models through influencing the central arginine vasopressin (AVP) system. Li et al. (2022) demonstrated that DSS pretreatment can restore the impaired abilities of rats caused by ETM-induced depression- and anxiety-like behavior. Although the antidepressant effect of DSS has been discovered, the functional components and underlying mechanisms are still unclear at a systematic and comprehensive level.

The complex multicomponents and multi-targets in TCM hinder our ability to identify the functional components and molecular mechanisms of its efficacy. Systems pharmacology, a promising holistic strategy for the research of TCM, can comprehensively elucidate the synergistic mechanism of the TCM formula by generating complicated component–target and target–disease interaction networks (Zhang et al., 2019; Correia et al., 2022). In recent years, with the development of computer technology and its mathematical algorithms, the computational systems pharmacology algorithm exhibits some advantages in analyzing the functional components and action mechanisms of TCM (Azer et al., 2021). For example, Wang et al. (2020) applied Huffman encoding and the random walk algorithm to uncover the action mechanisms of different prescriptions in treating rheumatoid arthritis. Gao et al. (2020) optimized components and explored underlining mechanisms of Lang Chuang Wan for treating systemic lupus erythematosus based on the contribution index formula. Yang et al. (2021) decoded the potent combination therapeutic mechanisms of Erxian decoction for osteoporosis on a novel system pharmacology model (Yang et al., 2021). The computer mathematical algorithm plays an important role in meeting the data dependence requirements of all aspects of systems pharmacology of TCM.

In this study, a novel computational systems pharmacology mathematical algorithm was designed and applied to detect the functional components group (FCG) and elucidate the therapeutic mechanisms of DSS in treating depression. First, all components in DSS were collected from databases and literatures. The active components were extracted from all components of DSS based on the criteria that combined Lipinski’s rule and Caco-2, and the component–target (C-T) network was constructed by predicting the targets of these active components based on published prediction tools. Second, the pathogenetic genes were collected from published databases and mapped to the protein–protein interaction (PPI) network to construct a weighted gene regulatory network of depression. The C-T network and pathogenetic gene regulatory network were combined to construct a C-T-P-D network. A network motif detection algorithm was designed and applied to capture the core intervention motif (CIM). The proteins in the CIM were defined as effective proteins, which could be used to screen effective components. Then the effective components in DSS were optimized and the FCG was obtained through the target coverage accumulation (TCA) algorithm, which would be selected to elucidate the underlining mechanisms of DSS in treating depression. Finally, a key component in FCG of DSS was used to perform experimental verification *in vitro and in vivo*.

## Materials and methods

### Construction of weighted gene regulatory network of depression

A comprehensive weight gene network of depression was constructed by the PPI data acquired from public databases, including HPRD, MINT, STRING, Reactome, BioGRID, DIP, and intAct. Depression-related genes were extracted from GeneCards (https://www.genecards.org). The genes with relevance scores higher than the average score were kept as high pathogenicity genes and then mapped to the PPI network, followed by the construction of the weighted gene regulatory network of depression. Cytoscape software (Version 3.7.0) was employed to visualize the network.

### Potential active component selection and target prediction

The components of DSS were acquired from published databases, followed by the selection of potential active compounds according to Lipinski’s rule and human intestinal cell line Caco-2. The prediction tools including HitPick, SwissTargetPrediction, and similarity ensemble approach (SEA) were employed to acquire the targets of active components from DSS. The detailed method of chemical components collection and target prediction is described in the Supporting Information.

### Network construction

Cytoscape software (Version 3.7.0) was used to create component–target (C-T) networks of DSS. Topological parameters of the networks were calculated by using NetworkAnalyzer.

### Core function motifs detection

Mining motifs in complex networks can help us to have a deeper understanding of the treatment of diseases by drugs. In order to decode core intervention motifs of DSS in treating depression, refer to our previous mathematical algorithm with slight modification (Wang et al., 2020):

$$q_{i↷}=\tau \frac{n-n_{i}}{n}\underset{\alpha \in i}{\sum }p_{\alpha \;}+\;\left(1-\tau \right)\underset{\alpha \in i}{\sum }\underset{\beta \notin i}{\sum }p_{\alpha }\omega_{\alpha \beta },$$

where $q_{i↶}$ is the probability of jumping out of Motif *i* and $\omega_{\alpha \beta }$ is the normalized weight for jumping from node *a* to node *ß*.

### Exploitation of the traditional chinese medicine algorithm to screen functional components group

In order to figure out the FCG in CIM, we assigned the target coverage of each component *t* in CIM as $H_{t}$ . The contribution index of targeted to pathogenic genes is $L_{t}$ . The expected maximum target coverage index of FCG in CIM is S. In these variables, S>0; FCG is required to be found from n components so that the cumulative contribution index of FCG targeted to pathogenic genes should have the maximum value. The detail calculation process is described as follows:

$$CCI=\max\overset{n}{\underset{t=1}{\sum }}L_{t}y_{t}\;\left(L_{ti}>0,\;1\leq t\leq n\right)\;\overset{n}{\underset{t=1}{\sum }}H_{t}y_{t}\leq S\;\;y_{t}\in \left\{0,1\right\},\;1\leq t\leq n\;\;\left(S>0,\;H_{t}>0\right)$$

Set the sub-problems of the given question as follows:

$$CCI_{sub}=\max\overset{n}{\underset{u=1}{\sum }}L_{u}y_{u},\;\;\;\;\;\overset{n}{\underset{u=1}{\sum }}H_{u}y_{u}\leq S_{sub}\;\;y_{u}\in \left\{0,1\right\},\;1\leq u\leq n.$$

$\text{m}\left(\text{t},\;C_{sub}\right)$ is the optimal solution when the expected network coverage is $S_{sub}$ and the optional component is q. From the optimal substructure properties, the recursive formula for calculating $\text{m}\left(\text{t},\;C_{sub}\right)$ can be established as follows:

$$m\left(t,\;S_{sub}\right)=\{\begin{array}{c} max\left\{m\left(t+1,\;S_{sub}\right),\;m\left(t+1,\;S_{sub}-H_{t}\right)+L_{t}\right\}\;S_{sub}\geq H_{t},\\ m\left(t+1,\;S_{sub}\right)\;\;0\leq S_{sub}<H_{t} \end{array}$$

$$m\left(n,\;S_{sub}\right)=\{\begin{array}{c} L_{n}\;S_{sub}\geq H_{n},\\ 0\;\;0\leq j<H_{n}. \end{array}$$

### Gene ontology and pathway analysis

A GO analysis was performed by using the clusterProfiler package of R software. The KEGG database was used to perform KEGG pathway analyses. The detailed methods of GO and pathway enrichment analyses are described in the Supporting Information.

### Experimental validation *in vitro* and *in vivo* and statistical analysis

The neuroprotective effect of the top three components including vanillic acid, anisic acid, and ferulic acid (20 μmol/L) in FCG of DSS was assayed on corticosterone-treated PC12 cells by the MTT assay with desipramine (20 μmol/L) as the positive control group. Ferulic acid, which exhibited a potent neuroprotective effect on corticosterone-treated PC12 cells, was employed to evaluate the possible mechanisms by using apoptosis assay and Fluo-4/AM staining assay. Hoechst 33342 and PI double fluorescent staining were used to evaluate the anti-apoptotic activity. Fluo-4/AM staining was further performed to observe the effect of FA on the concentration of intracellular Ca^2+^ ([Ca^2+^]i).

In addition, the forced swimming test (FST) and the tail suspension test (TST) models were used to verify the antidepressant effect of FA. A total of 50 male ICR mice were randomly divided into five groups as follows (*n* = 10): control group, FA (25 mg/kg) group, FA (50 mg/kg) group, FA (100 mg/kg) group, and venlafaxine (50 mg/kg) group. The drug was dissolved in normal saline with DMSO less than 0.1% [v/v] (vehicle). The solutions of drugs were administered to the mice *via* gastric intubation at a dosage of 0.2 ml/10 g (body weight) once daily between 9:00 a.m. to 10:00 a.m. for 14 days. Simultaneously, mice in the control group were orally given a vehicle at an equal volume. The study was approved by the Experimental Animal Ethical Committee of Modern Research Center for Traditional Chinese Medicine, Shanxi University. All experimental procedures in this study were carried out in accordance with the NIH Guide for the Care and Use of Laboratory Animals.

SPSS 19.0 software was used to conduct a statistical analysis. The detailed methods of experiment and the statistical analysis are described in the Supporting Information.

## Results

**FIGURE 1:**
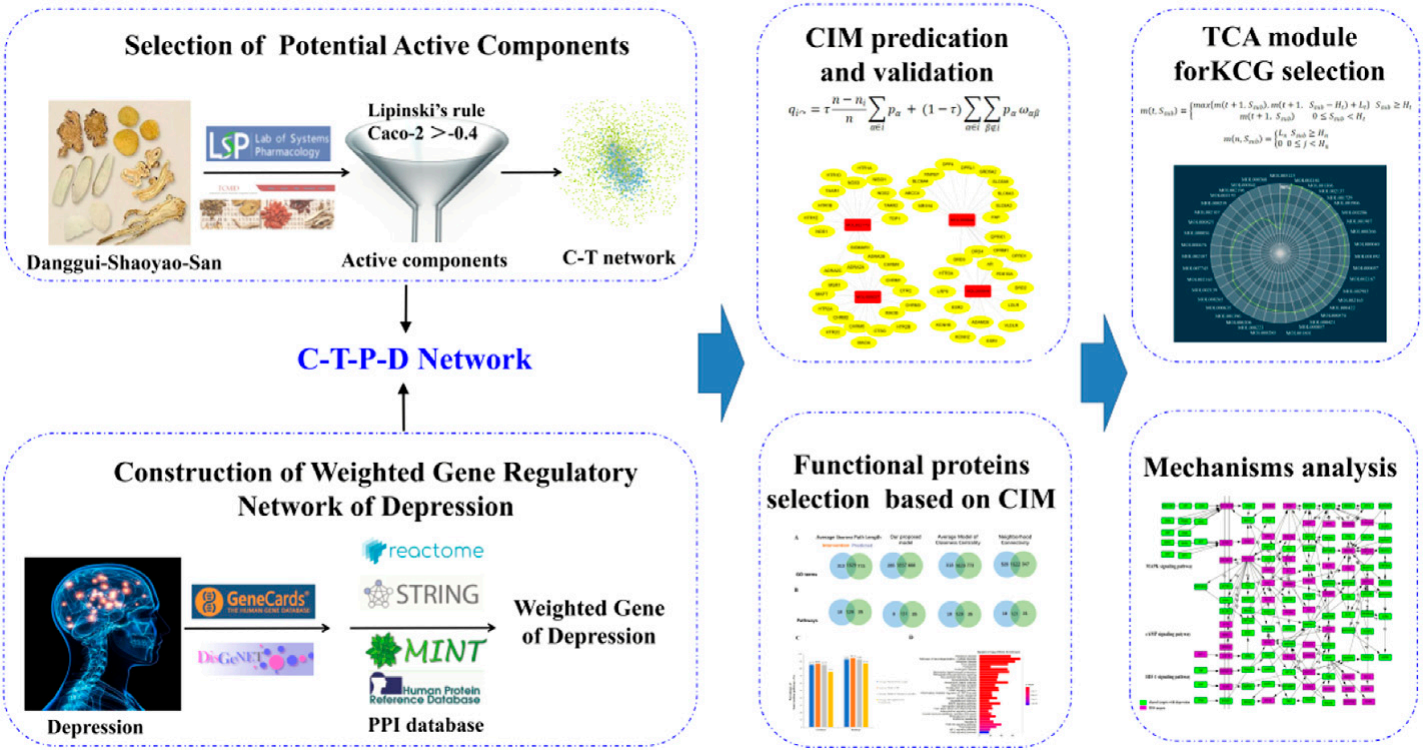
Flowchart of our proposed computational systems pharmacology approach.

In this study, a novel computational systems pharmacology algorithm was designed and applied to detect the functional components group and explore the therapeutic mechanisms of DSS in the treatment of depression. The workflow of computational systems pharmacology approach is demonstrated in Figure 1 and described as follows: 1) all components in DSS were collected from databases and literatures; 2) the active components were extracted based on the criteria that combined Lipinski’s rule and Caco-2, and the C-T network was constructed; 3) the pathogenetic genes were collected from publish databases and used to construct the weighted gene regulatory network of depression through mapping to PPI; 4) the C-T and pathogenetic gene regulatory networks were combined to construct the C-T-P-D network; 5) a network motif detection algorithm was designed and applied to capture CIM; 6) the proteins in the CIM were defined as effective proteins, which could be used to filter effective ingredients; 7) the TCA algorithm was employed to optimize effective ingredients and obtain the FCG, and 8) the FCG was used to uncover the underlining mechanisms of DSS in the therapy of depression.

### Chemical analysis

The concentration of 13 components from DSS were collected based on the published literature (Chen et al., 2009) and then demonstrated in Table 1. The content of the constituents including ferulic acid, coniferyl ferulate, levistolide A, Z-butylidenephthalide, senkyunolide I, gallic acid, albiflorin, paeoniflorin, senkyunolide A, 3-butylphthalide, Z-ligustilide, atractylenolide II, and atractylenolide I will provide an experiment-aided chemical space for searching the effective ingredients and provide support for further analysis.

**TABLE 1:** Information on the chemical analysis of DSS from the literature. DSS, Danggui-Shaoyao-San.

Herb	Component	Concentration (mg/g)
Angelica Sinensis Radix (Danggui)	Ferulic acid	0.25
	Coniferyl ferulate	0.64
	Levistolide A	0.26
	*Z*-Butylidenephthalide	0.16
	Senkyunolide I	0.90
Paeoniae Radix Alba (Baishao)	Gallic acid	0.48
	Albiflorin	1.86
	Paeoniflorin	2.69
Chuanxiong Rhizoma (Chuanxiong)	Senkyunolide A	2.14
	3-Butylphthalide	0.35
	*Z*-Ligustilide	2.54
Atractylodis Macrocephalae Rhizoma (Baizhu)	Atractylenolide II	0.01
	Atractylenolide I	0.04

### Selection of potential active components

A total of 526 components were obtained from six herbs in DSS by searching in the TCMSP, TCMID, and TCM@ Taiwan databases. As a result, 336 active ingredients in DSS, which passed the filtering criteria that combined the Lipinski’s rule and Caco-2, were obtained after removing the duplicate components. The detail information of these ingredients is shown in Supplementary Table S1.

### Construction of weighted gene regulatory network of depression

**FIGURE 2:**
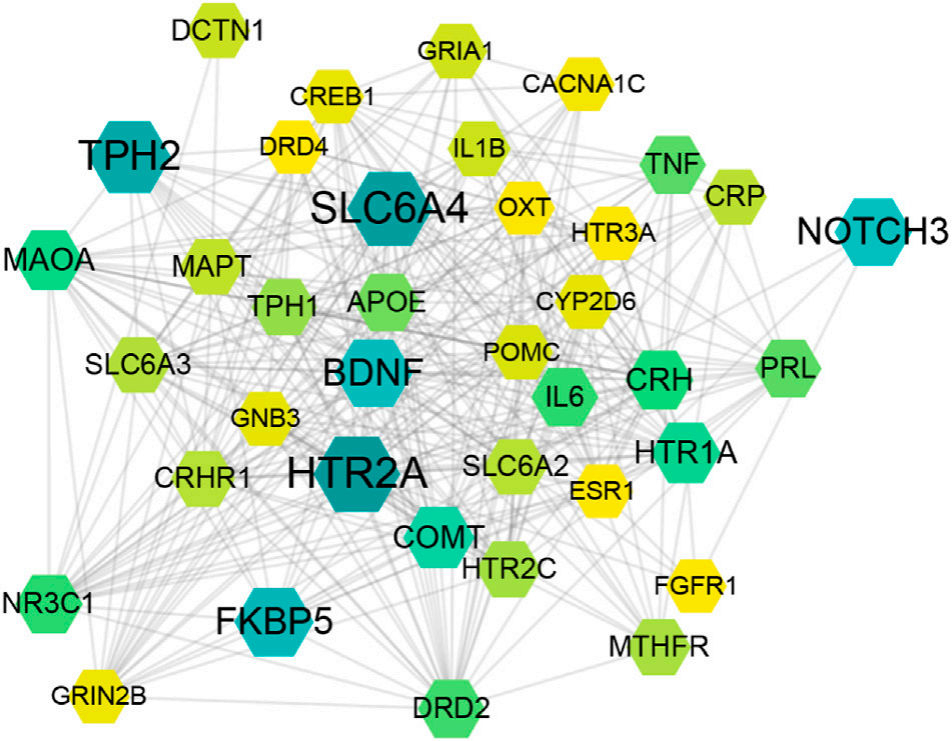
Disease weight gene regulatory network of depression. The size and color of the node represents the relevance score of targets.

For understanding the pathogenesis of depression and provide therapeutic strategies, we erected and analyzed the weighted gene regulatory network of depression. Depression-related genes were obtained from the GeneCards database. A total of 3,713 genes were collected as depression-related genes (Supplementary Table S2). The public databases (MINT, STRING, Reactome, HPRD, DIP, BioGRID, and intAct) were employed to set PPI data, which was further applied to construct a comprehensive weighted gene network of depression. These genes were further used to construct the weighted gene regulatory network of depression through mapping to the PPI network, which contains 3,039 nodes and 42,970 edges (Figure 2). Genes with higher weights include SLC6A4, BDNF, HTR2A, TPH2, COMT, CRH, IL6, and TNF. HTR2A encodes one of the receptors for serotonin, a neurotransmitter with many roles, which play an important role in major depressive disorder (Marshe et al., 2017). SLC6A4’s primary function in the central nervous system involves the regulation of serotonergic signaling *via* transport of serotonin molecules from the synaptic cleft back into the pre-synaptic terminal for re-utilization (Park et al., 2019). These genes are involved in the complex pathological mechanism of depression, including the neurotransmitter system (e.g., HTR2A and SLC6A4), neurotrophin (e.g., BDNF), hypothalamic-pituitary-adrenal (HPA) axis (e.g., CRH), and inflammation (e.g., IL6, TNF). These results demonstrate that the regulatory network of weight genes can explain the pathogenesis of depression, which will provide the basis for the construction the function motifs.

### C-T-P-D network construction

In order to promote the analysis of the complex relationships between active ingredients of DSS and their targets, Cytoscape was used to construct component–target networks (Supplementary Figure S1). The results showed that 336 active ingredients, 1,443 target proteins, and 11,828 interactions were observed in the C-T network. The NetworkAnalyzer was further used to analyze the topology parameters of the C-T network, and the result demonstrated that the average degree of active ingredients and their targets were 34.79 and 8.20, respectively. The phenomena that interactions between one constituent and multiple targets and different ingredients acting on a single target were observed in the C-T network, which coincides with the viewpoint of “multicomponent and multi-target” of TCM.

We constructed the C-T-P-D network based on the active components targets network and the weighted gene regulatory network. The network contains 4,278 nodes and 77,463 edges. Based on the network analysis, the network topology attribute analysis of the network model was performed. As a result, the network heterogeneity and centralization were 1.344 and 0.163, respectively. Degree and betweenness of node were regarded as the most critical indicators of the network analysis. The value of degree and betweenness represented the importance of node. According to the results of the network analysis, four targets with higher degrees and betweenness values, including ESR1 (degree: 411; betweenness: 0.02458607), MAPT (degree: 253; betweenness: 0.02371366), AKT1 (degree: 153; betweenness: 0.00357142), and ALB (degree: 79; betweenness: 0.00317278) were probably the key targets of DSS in the treatment of depression.

### Core intervention motif predication and validation

**FIGURE 3:**
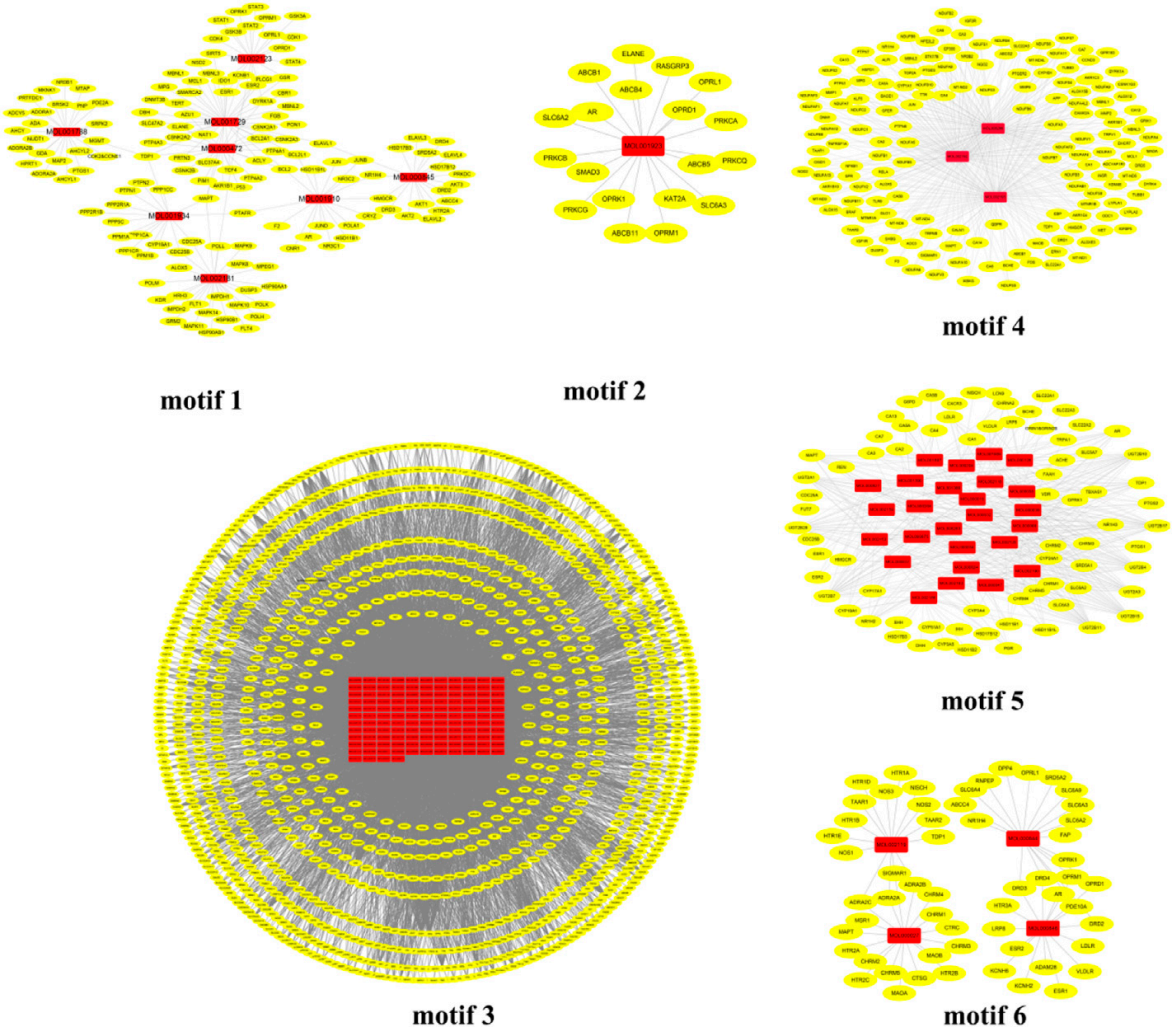
Predicated CIM of the C-T network of DSS. The red nodes represent the specific components of DSS, and the yellow nodes represent related targets. CIM, key core function motifs; C-T, component–target.

According to the random walk theory and Huffman encoding, the infomap algorithm was introduced to the network pharmacology model to extract critical information from the C-T-P-D network and explore the potential molecular mechanisms. The algorithm was performed to optimize the discovery of core intervention motifs in the C-T network through a reasonable global metric. As a result, six core intervention motifs were predicted in DSS (Figure 3). The detail information of network core intervention motifs is demonstrated in Supplementary Table S3.

**FIGURE 4:**
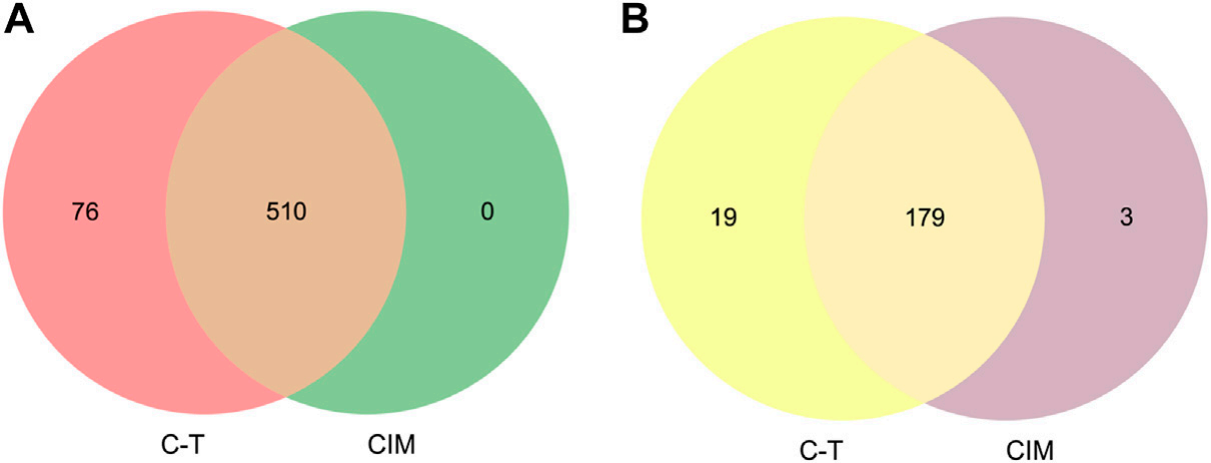
Venn diagram shows the overlap number of pathogenic genes between the C-T network and CIM **(A)**. Functional similarity analysis between the C-T network and CIM **(B)**. CIM, key core function motifs; C-T, component–target.

In order to validate whether predicted CIM can represent full C-T network, two strategies were applied to certify the reliability and accuracy of CIM. First, the overlap of pathogenic genes’ number between CIM targets and C-T network targets were compared. We found that the C-T network contains 586 pathogenic genes. CIM contain 510 pathogenic genes (Figure 4A). Second, the gene enrichment pathways in CIM and the C-T network were compared. The results showed that among the 198 pathways enriched by the target genes of the CIM, 179 coincided with the enriched pathways of pathogenic genes, accounting for 90.40% of the gene enrichment pathway of the C-T network (Figure 4B). The aforementioned results confirmed that a high coincidence degree was observed in CIM and the C-T network in the coverage of pathogenic genes and the gene functional level, which also provides a basis for subsequent functional protein selection.

### Functional protein selection and validation based on core function motifs

**FIGURE 5:**
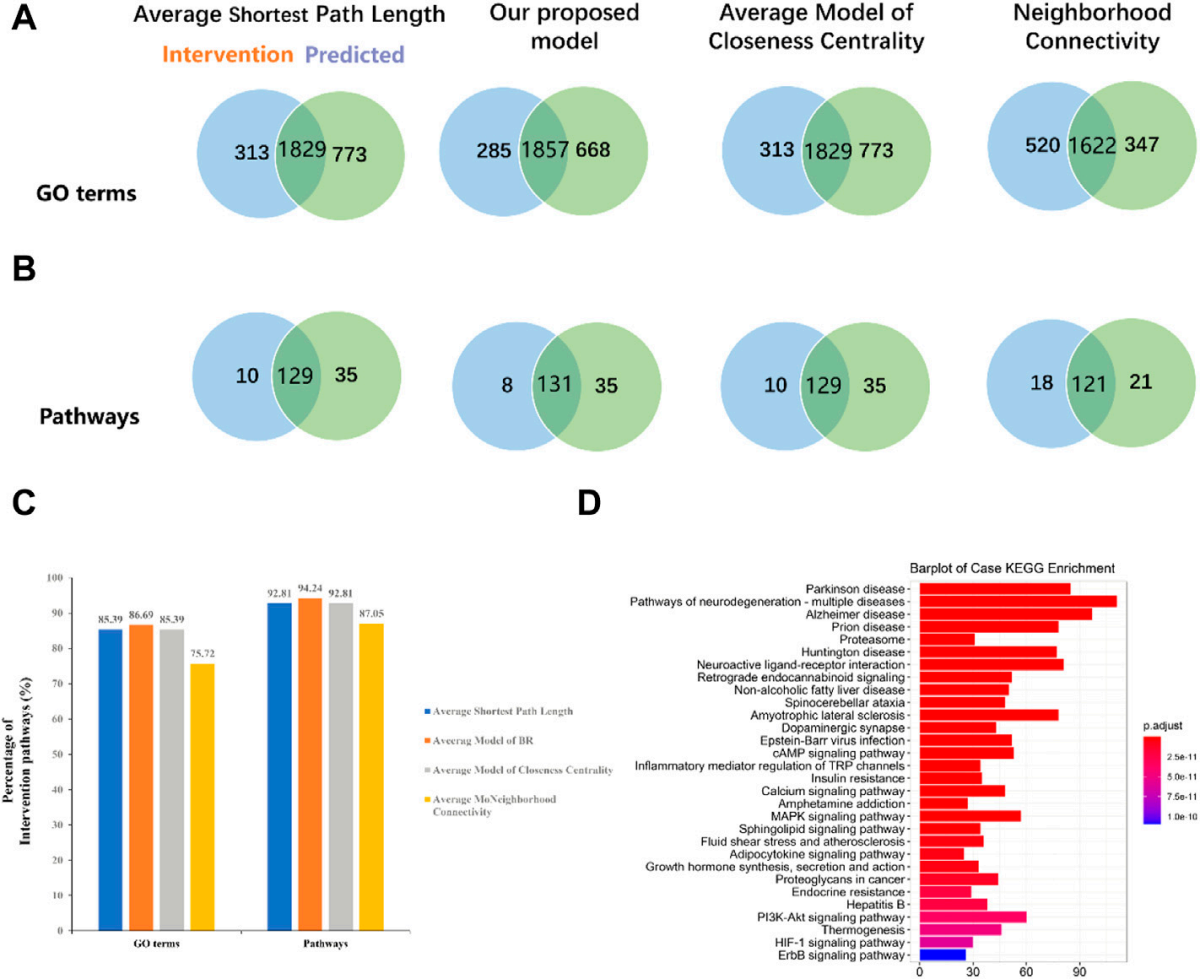
Compared our proposed model with other widely used models. **(A)** Venn diagrams display overlapping GO terms of four models with intervention GO terms. **(B)** Venn diagrams display overlapping pathways of four models with intervention pathways. **(C)** Comparison of our model with other models on the intervention pathways and GO terms. **(D)** Bar plot for effective proteins’ pathway enrichment analysis.

One of the most important topological properties that could be employed to optimize the network is the importance of nodes in a network. In the current study, a novel approach to calculate the importance of nodes was designed, which takes into account the influence and connectivity of nodes. Based on our method, the pathway and GO term analyses of 580 effective proteins account for 94.24% and 86.69% of the intervention pathways and GO terms, respectively (Figures 5A–C). Compared with the average shortest path length, closeness centrality, and neighborhood connectivity, the percentage of the intervention pathway and the intervention GO term in our model was significantly increased, suggesting that our model has higher precision and higher functional coverage.

The results of pathway enrichment showed that these effective proteins participated in the cAMP signaling pathway (hsa04024), calcium signaling pathway (hsa04020), and PI3K-Akt signaling pathway (hsa04151) (Figure 5D). Research works have indicated that the cAMP signaling pathway is associated with the pathophysiology of depression and cognitive function impairments (Cao et al., 2018). Dysfunction of the cAMP-mediated cascade, such as decreased G protein and cAMP levels, reduced adenylyl cyclase (AC) and protein kinase A (PKA) activity, and altered PKA-mediated phosphorylation, have been observed in depression patients (Shelton et al., 1996). Further study has suggested that chronic antidepressant treatment could upregulate cAMP signal transduction and PKA activity in the brain (Wang et al., 2018). Abnormal neuronal calcium homeostasis and calcium signaling pathway have been postulated to be involved in depressive symptoms and cognitive deficits (Grützner et al., 2018). Calcium participates in the development of neurons, and the overloading of intracellular Ca^2+^ could result in neuronopathy, which facilitates the development of depression (Meldrum, 1986). In addition, the PI3K/Akt signaling pathway was also associated with neurobiology of depression and modulated by pharmacological antidepressant strategies (Ludka et al., 2016).

### FCG selection and validation

**FIGURE 6:**
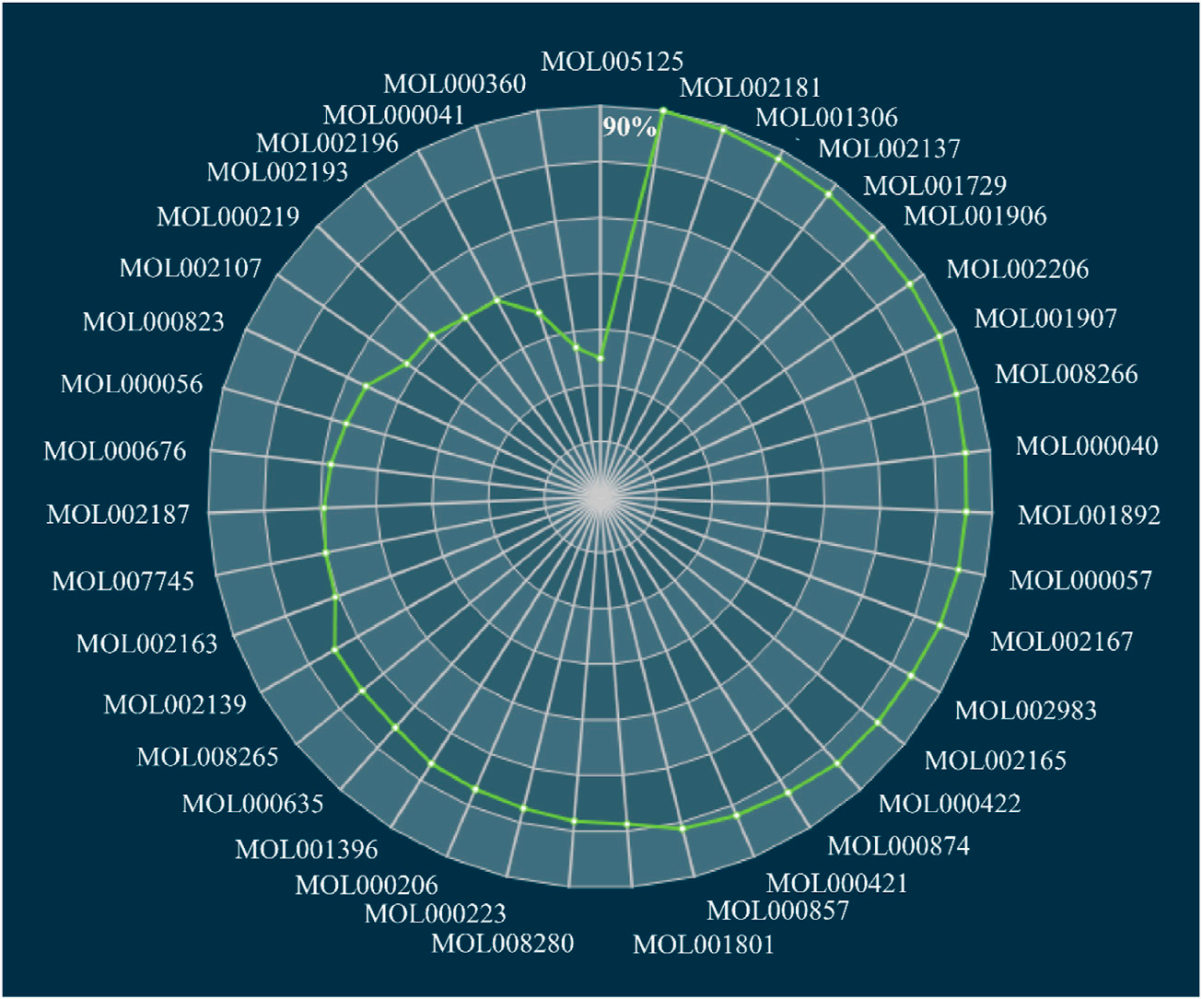
Accumulative FCG score of active components in DSS. FCG, functional components group; DSS, Danggui-Shaoyao-San.

In the current study, we established a TCA algorithm to optimize CIM and obtain the FCG, which would be further employed to speculate the molecular mechanism of DSS in the treatment of depression. The contribution accumulation results demonstrated that the top five components including vanillic acid (DSS144), anisic acid (DSS59), ferulic acid (DSS17), phenylalanine (DSS297), and hexaphenone (DSS269) contribute to 51.03% target coverage of effective proteins. In addition, 40 components accounting for 90.00% targets coverage of effective proteins were grouped as FCG (Figure 6). Higher targets coverage rate of the effective proteins demonstrated that the FCG selected from DSS played a leading part in the therapy of depression.

**FIGURE 7:**
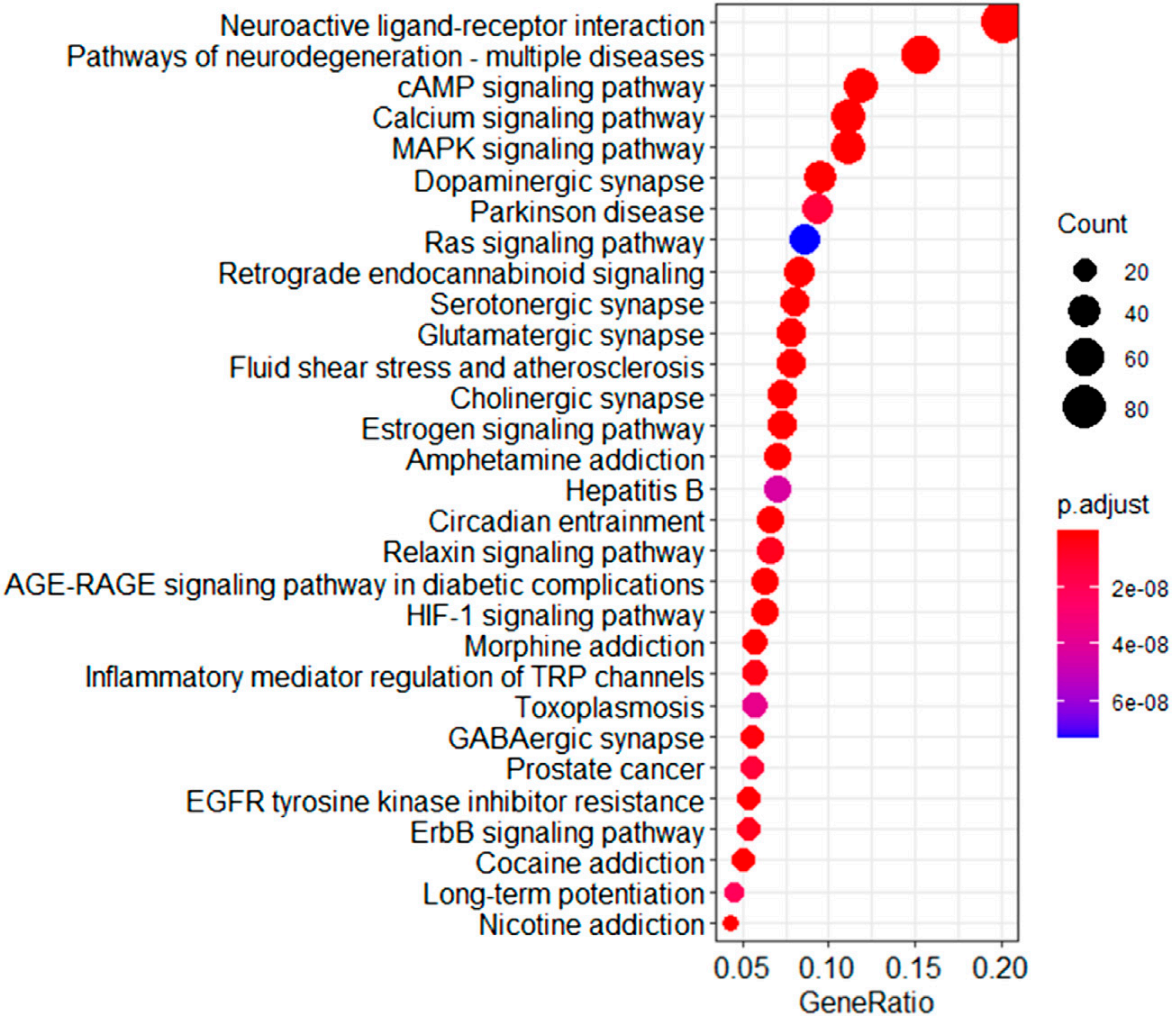
Pathway enrichment analysis of the targets of FCG in DSS. FCG, functional components group; DSS, Danggui-Shaoyao-San.

A pathway analysis was performed using FCG targets and depression pathogenic genes to analyze the treatment of depression by DSS at a functional level. The results showed that the number of pathways enriched by FCG targets was 169 (*p* < 0.05), and the number of pathways enriched by pathogenic genes was 208 (*p* < 0.05), which covered 81.25% of the pathogenic genes enriched pathways. The major targets of FCG were frequently enriched in the cAMP signaling pathway (hsa04024), calcium signaling pathway (hsa04020), MAPK signaling pathway (hsa04010), Ras signaling pathway (hsa04014), estrogen signaling pathway (hsa04915), and HIF-1 signaling pathway (hsa04066) (Figure 7). The result showed that the approach based on CIM and the TCA algorithm for optimizing the TCM formula is reliable.

### GO enrichment analysis of FCG targets

**FIGURE 8:**
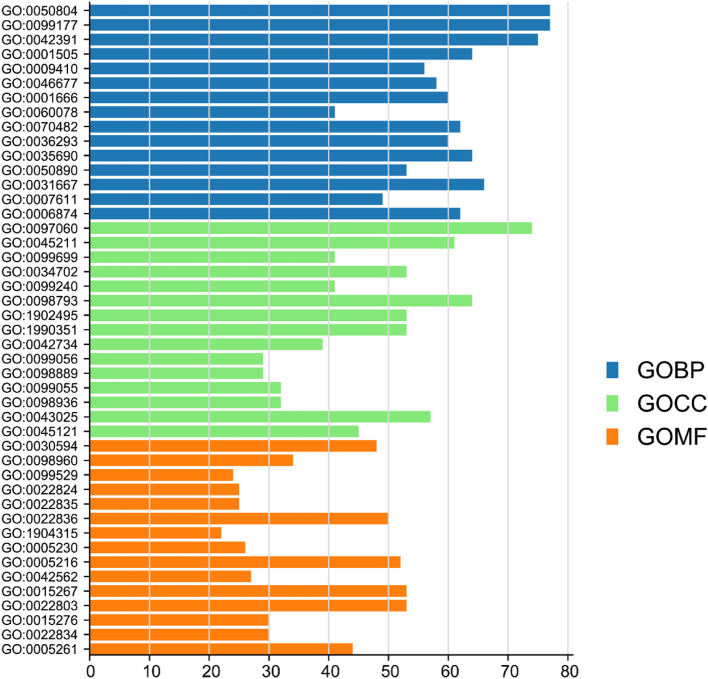
Go enrichment analysis of the targets of FCG. FCG, functional components group.

In order to further expound the mechanism of DSS, a GO enrichment analysis was performed to enrich all the targets interacting with FCG of DSS (Figure 8). The results showed that the targets regulated by DSS were enriched in biological processes related to neurotransmitter transmission and response to oxygen. For example, the pathways of neurotransmitter transmission are modulation of chemical synaptic transmission (GO:0050804, ABAT, BCHE, CACNA1A, and DLG4), regulation of membrane potential (GO:0042391, AKT1, BCL2, CFTR, and GABBR1), regulation of neurotransmitter levels (GO:0001505, ACHE, CNR1, HRH3, and MAOA), and regulation of postsynaptic membrane potential (GO:0060078, CHRM1, DRD2, GRIA1, and GSK3B). Important genes involved in neurotransmitter transmission in these pathways include ACHE, MAOA, and DRD2 (Zheng et al., 2016; Youdim, 2018; Belkacemi and Darmani, 2020) are related to the development of depression. The pathways of response to oxygen are response to hypoxia (GO:0001666, ABAT, APAF1, MECP2, and MMP2), response to oxygen levels (GO:0070482, AKT1, HMOX1, MYC, and NOS1), and response to decreased oxygen levels (GO:0036293, CASR, CAT, PTGS2, and RHOA). Hypoxia is a common finding in depression, which further influences the synthesis of neurotransmitter serotonin and finally accelerates the development of depression (Østergaard et al., 2018). The GO analysis suggested that DSS treat depression through regulation of neurotransmitter transmission and response to oxygen.

DSS regulates the GO cellular component of depression, including synaptic membrane (GO:0097060, ADORA1, CACNA1D, DRD1, and GABBR2), ion channel complex (GO:0034702, ABCC9, CHRNA1, GABRA1, and KCNA1), transporter complex (GO:1990351, CFTR, GRIA1, PDE4B, and TRPC3), and neuronal cell body (GO:0043025, BACE1, HTR2A, PTPRF, and SIRT2). Synaptic dysfunctions play important roles in the pathogenesis of depression, including the disruption in structural and molecular level (Calabrese et al., 2016). In addition, the correlation between depression and neural plasticity has been reported in numerous clinical and basic research studies (Liu et al., 2017). Our results suggested that DSS may play a part in the treatment of depression by modulating targets on the synapsis neuronal cell body.

DSS regulates the GO molecular function of depression including neurotransmitter receptor activity (GO:0030594, DRD1, GABBR1, GRIA1, and HTR2A), ion channel activity (GO:0005216, CACNA1A, GRIA1, SCN2A, and TRPC3), and passive transmembrane transporter activity (GO:0022803, ABCC9, BCL2, CFTR, and CNR1). Our results indicated that DSS may affect autoreceptors and transporters of neurotransmitters to regulate neurotransmitter’s homeostasis in the synaptic cleft, which was closely related to the pathogenesis of depression (David and Gardier, 2016).

### Potential mechanism analysis of Danggui-Shaoyao-San in treating depression

**FIGURE 9:**
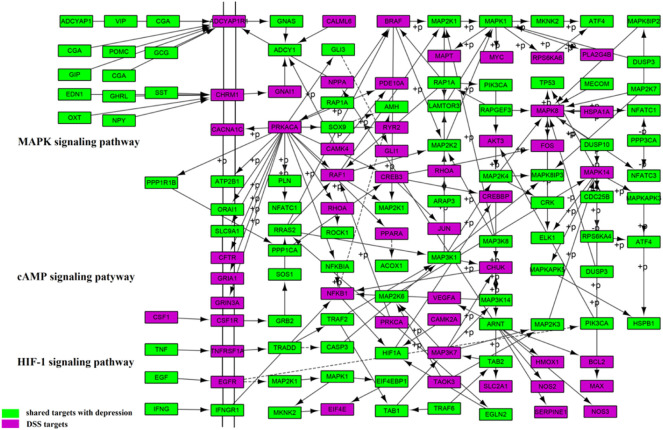
Distribution of targets of FCG in the comprehensive signaling pathway. FCG, functional components group.

In order to illustrate the mechanisms of DSS in the treatment of depression at the system level, three important molecular pathways including the MAPK signaling pathway (hsa04010), cAMP signaling pathway (hsa04024), and HIF-1 signaling pathway (hsa04066) were used to construct a comprehensive signaling pathway (Figure 9). By searching from PubMed literature, the three pathways were confirmed to play important roles in the development of depression.

The first five columns were considered as upstream and the rest columns were considered as downstream position of the pathway. Among these pathways, the MAPK signaling pathway (hsa04010) is regarded as one of the most important pathways in the treatment of depression with DSS. As demonstrated in Figure 9, DSS may regulate ADCYAP1R1 protein through the upstream ADCYAP1, CGA, and POMC and regulate CHRM1 protein through the upstream EDN1, GHRL, SST, and OXT. In addition, it is shown that DSS can also regulate a key protein RAP1A in the MAPK signaling pathway, resulting in the influence of BRAF, MAPK1, PIK3CA, and PRKACA cascade.

The cAMP signaling pathway (hsa04024) and the HIF-1 signaling pathway (hsa04066) were also important pathways in the treatment of depression by DSS. A total of 47 targets were located in the cAMP signaling pathway, such as CSF1, GRB2, CREB3, and JUN. A total of 25 targets were located in the HIF-1 signaling pathway, such as TNF, EGF, HIF1A, and TRAF2. DSS may affect downstream of NFKB1 protein through the upstream IFNG and IFNGR1, followed by the regulation of downstream HIF1A cascade. In addition, TRADD could be regulated by DSS through the upstream TNF and TNFRSFIA, resulting in downstream TRAF2, TAB1, and MAP2K3 cascade amplification. The results suggested that DSS plays a therapeutic role by targeting different genes in the comprehensive pathway.

### Experimental validation *in vitro* and *in vivo*

In order to further verify the results obtained by the network pharmacology analysis, the top three components including vanillic acid, anisic acid, and ferulic acid (20 μmol/L) in FCG of DSS were selected for experimental validation on PC12 cells by the corticosterone-induced damage model. As a result, desipramine (positive control), vanillic acid, anisic acid, and ferulic acid exhibited moderate neuroprotective activities to improve the PC12 cell survival rates from 56.34% ± 1.31% to 70.48% ± 1.02%, 59.68% ± 0.71%, 62.09% ± 2.53%, and 68.01% ± 1.72%, respectively. In addition, compared with vanillic acid and anisic acid, ferulic acid exhibited better neuroprotective activity (*P* < 0.01, Table 2). Therefore, ferulic acid was selected for further study.

**TABLE 2:** Protective effect of compounds on corticosterone-induced PC12 cells.

Group	Concentration (μmol/L)	Cell survival rate (% of control)
Control	0	100.00 ± 1.72
Corticosterone	400	56.34 ± 1.31^##^
Desipramine	20	70.48 ± 1.02**
Vanillic acid	20	59.68 ± 0.71*^△△^
Anisic acid	20	62.09 ± 2.53**^△△^
Ferulic acid	20	68.01 ± 1.72**

^##^ *P* < 0.01, compared with the control group, **P < 0.01, compared with the corticosterone-treated group, ^△△^ *P* < 0.01, compared with the ferulic acid group. One-way analysis of variance was used, *n* = 3.

**FIGURE 10:**
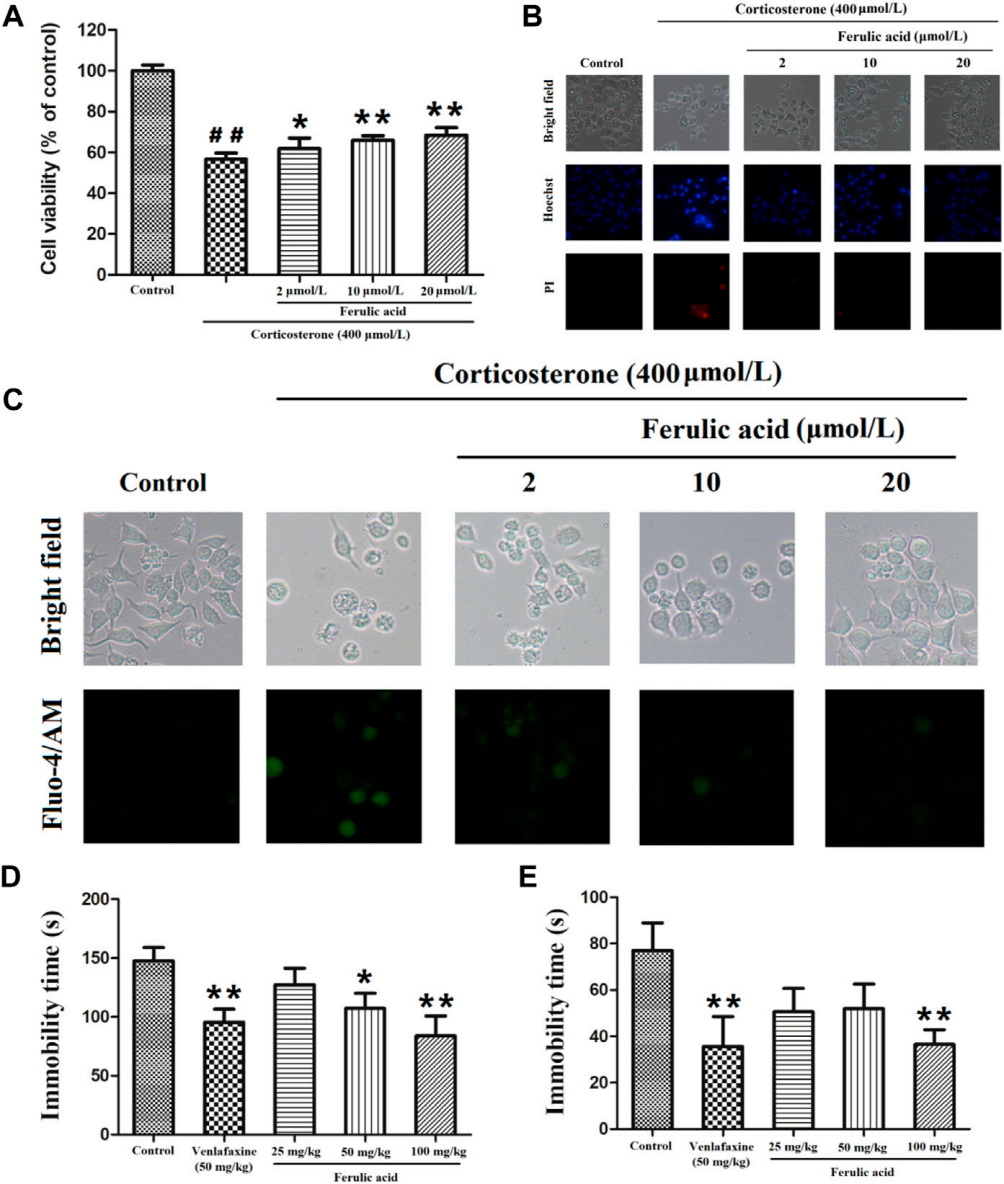
Antidepressant effect validation of a key component (FA) in FCG of DSS. **(A)** Protective effect of FA on corticosterone-induced PC12 cells (*n* = 4). ^##^ *P* < 0.01, compared with the control group; *P < 0.05 or **P < 0.01, compared with the corticosterone-treated group; **(B)** cell survival analyzed by Hoechst 33342 and PI double staining; **(C)** representative Fluo-4/AM staining images of intracellular Ca^2+^; **(D)** effect of FA on the immobility time in the forced swim test (*n* = 10); **(E)** effect of FA on the immobility time in the tail suspension test (*n* = 10). Drugs were orally administered once daily for 14 days; the tail suspension test was conducted at the 13th day, and the forced-swimming test was conducted at the 14th day. *P < 0.05, **P < 0.01, compared with the vehicle group.

As demonstrated in Figure 10A and Supplementary Table S5, compared with the control group, the cell viability was significantly decreased by 56.61% ± 3.01% in corticosterone-treated cells. However, FA (2, 10, and 20 μmol/L) markedly increased the cell viability by 61.80% ± 5.21%, 65.90% ± 2.26%, and 68.49% ± 3.61%, in a concentration-dependent manner. Hoechst 33342 and PI double staining assay was carried out to analyze the effect of FA on cell apoptosis. As depicted in Figure 10B, the proportion of apoptotic cells with nuclear fragmentation and condensation (Hochest33342, blue), as well as that of necrotic cells with the loss of cell membrane integrity, was obviously increased after corticosterone treatment. However, the treatment of FA (2, 10, and 20 μmol/L) significantly reduced the elevated apoptotic and necrotic cells induced by corticosterone.

According to the results of the pathway enrichment analysis, the calcium signaling pathway was involved in the antidepressant mechanism of FCG in DSS. Fluo-4/AM staining was further performed to observe the effect of a representative component (FA) on the concentration of intracellular Ca^2+^([Ca^2+^]i). The result is illustrated in Figure 10C. Compared with the control group, the green fluorescence in cells of the corticosterone-treated group was enhanced due to an increase of [Ca^2+^]i. However, pretreatment with FA could significantly reverse the enhanced green fluorescence, which indicated that the calcium signaling pathway was indeed related to the antidepressant mechanism of FCG in DSS.

The *in vivo* antidepressant effect of FA on two animal models of depression is shown in Figures 10D,E and Supplementary Tables S6, S7. Compared with the vehicle group, the immobility time in FST and TST was significantly reduced in the FA (50 and 100 mg/kg) and venlafaxine (50 mg/kg) treatment groups. Our results demonstrated that FA possesses potential antidepressant effect.

## Discussion

At present, western medicines are the main antidepressants commonly used in clinics, but there are many problems, such as many adverse reactions, slow onset, and poor curative effect. The TCM formula can achieve the same curative effect as western medicines in treating depression (Li et al., 2020). The TCM formula can not only effectively improve patients’ depression symptoms but also treat different symptoms of primary disease and concomitant disease, which can better maintain patients’ mental health. In the treatment of depression, the multicomponent, multi-target, and multilevel mode of action of the TCM formula has played a great role. At the same time, it also brings great challenges to analyze the mechanisms of action of the TCM formula. How to identify the most effective intervention relationship from the complex network of treating depression with the TCM formula is the key to discover the functional components and action mechanisms of the TCM formula, and it is also the basis of the second excavation of the TCM formula.

By constructing a “drug–target–disease” network, systems pharmacology explores the interaction between biological functions and diseases at each node of the network, which is in line with the therapeutic principles of the TCM formula. Systems pharmacology is a powerful tool widely used to predict the potential mechanisms of TCM for treating diseases. However, there are few research reports on optimization of the TCM formula based on systems pharmacology. With the rapid development of computer technology, mathematical algorithms, and bioinformatics, our study proposes a comprehensive computational systems pharmacology model to optimize the TCM formula based on the mathematical algorithm. According to this model, we capture the CIM of DSS in treating depression and trace FCG from the CIM to speculate the possible mechanism of DSS in treating depression. This method provides a methodological reference for the interpretation of molecular mechanisms of TCM in the treatment of diseases and the development of new drugs.

We used a network motif detection model to predict the CIM and performed a functional analysis and verification. The result demonstrated that the C-T network contained 586 pathogenic genes and the CIM contained 510 pathogenic genes. A further pathway enrichment analysis demonstrated that among the 198 pathways enriched by the target genes of the CIM, 179 coincided with the enriched pathways of pathogenic genes, accounting for 90.40% of the gene enrichment pathway of the C-T network. The result suggested that we have deleted the invalid or weak effect relationships and increased the functional coverage rate after optimization. Next, the TCA algorithm was applied to optimize effective ingredients and obtain the FCG. The FCG comprised 40 components from DSS, and the pathways enriched by the target genes of FCG were selected to elucidate the potential mechanism of DSS for the treatment of depression.

Our proposed screening strategy possesses two advantages. The first level of design is to construct function motifs based on the infomap algorithm and verify that the information of disease-related genes will be preserved as much as possible in this step as the core protein or functional protein in the motifs. In the second step, potential FCG are reversely screened based on the core proteins or functional proteins, and further pathway enrichment analysis is performed on the functional component group for verification. The results of this dual model and verification fully demonstrate the reliability of our integrated analysis model and provide a methodological reference for other prescription optimization.

Among the 40 key chemical compounds in FCG, the top five components including vanillic acid, anisic acid, ferulic acid, phenylalanine, and hexaphenone contribute to 51.03% target coverage of effective proteins, which play a critical role in the treatment of depression. Therefore, three accessible exogenous components including vanillic acid, anisic acid, and ferulic acid were used to evaluate the pharmacological effects in the model of corticosterone-treated PC12 cells. The results demonstrated that the neuroprotective activity of FA was superior to that of vanillic acid and anisic acid. Several studies have confirmed that FA is the major constituent in DSS, and the content of FA in DSS was much higher than that of vanillic acid and anisic acid (Chen et al., 2009; Bi et al., 2021). Therefore, FA was selected as a typical component for further experimental validation. However, the model of corticosterone-treated PC12 cells employed in the current study is based on the hypothalamic-pituitary-adrenal (HPA) axis dysfunction of the pathological mechanism of depression (Gong et al., 2019), while the pathogenesis of depression also involves monoaminergic mechanisms and neuroplasticity mechanisms (Dale et al., 2015). The result of cell experiment in the current study could not comprehensively reflect the antidepressant effects of these active ingredients. Vanillic acid (100 mg/kg) has also been reported to exert antidepressant effect in the forced swim test, and the mechanism was involved in the AMPAR–Akt–mTOR signaling transduction pathway (Chuang et al., 2020).

Ferulic acid (FA, 4-hydroxy-3-methoxycinnamic acid) is a natural phenolic acid abundant in TCM, including Angelica Sinensis Radix, Chuanxiong Rhizoma, and Ferula teterrima Kar. Et Kir. (Hassanzadeh and ArbabiAtyabi, 2017). In addition, it has been also found in fruits and vegetables such as tomatoes, oranges, apples, sweet corn, and rice bran (Mancuso and Santangelo, 2014). With the advantage of traversing the blood–brain barrier and reaching into hippocampus, FA has been confirmed to possess several neuropharmacological properties, such as antidepression (Wang et al., 2019). Sasaki et al. (2019) demonstrated that the underlying mechanisms of antidepressant-like effect of FA were involved in enhancing energy production. It has also been demonstrated that FA ameliorated the behavioral and oxidative stress alterations in the corticosterone chronic model, which was related to the repair of stress caused by the HPA axis dysfunction (Zeni et al., 2017). In the current study, FA has been proved to be a key component in FCG of DSS. Furthermore, the antidepressant effects and related mechanisms of the component was validated *in vitro and in vivo*.

Among the critical pathways enriched by FCG of DSS, the calcium signaling pathway is one of the most important pathways in the treatment of depression with FCG. A previous study has demonstrated that a dysregulation of the intracellular Ca^2+^ homeostasis (e.g., excess of intracellular Ca^2+^) contributes to the pathogenesis of depression (Bergantin, 2020; Kawatake-Kuno et al., 2021). Thus, the calcium signaling pathway was selected as a typical pathway for experimental validation in the current study. In the future, molecular biology experiments should be carried out to verify the effect of FCG on other signaling pathways such as cAMP signaling pathway, MAPK signaling pathway, and HIF-1 signaling pathway. In addition, the effect of FA on the calcium signaling pathway was preliminary verified based on corticosterone-treated PC12 cells in the current study.

In addition, this study inserted the TCA algorithm to screen FCG of DSS in treating depression on the basis of the original CIM detection using the infomap algorithm. The reason of amend this part of the algorithm was for searching functional components, while the previous algorithm only looked for network motifs. The benefits of these changes are that one can more accurately obtain the FCG of the TCM formula. The similarity of the two algorithms is that they can all obtain the functional motifs in the formula. The difference of the two algorithms is that the previous algorithm can better reflect the functional motifs of “treating the same disease with different treatments” and the cumulative contribution rate of these functional motifs, while the current algorithm is more inclined to obtain the FCG in the TCM formula. The accuracy of the two algorithms changes according to different purposes, but the accuracy of the current algorithm is also high after verification. There are similar algorithms here, such as Louvain and label propagation. In this study, the TCA algorithm is inserted into these algorithms and optimized again to achieve accurate FCG search for DSS treatment of depression.

However, the limitations of this study exist. First, this algorithm cannot predicate the activation or inhibition effects of the targets. Second, to evaluate the *in vivo* antidepressant effect of these key compounds, several animal models based on different pathogenesis of depression should be employed in the future research. Finally, more study on the *in vivo* effect of FA on the calcium signaling pathway would be useful to understand the possible antidepressant mechanisms of FA. We hope, in the future, to explore the effect of FA on the calcium signaling pathway by knocking out genes or blocking pathways based on patients or animal models of depression.

## Conclusion

Overall, a novel strategy to capture the functional components and mechanisms from TCM based on a mathematical algorithm was designed and applied to detect the FCG and decode the therapeutic mechanisms of DSS in treating depression. Therefore, the findings in this study provide a methodological reference for discovering functional components and interpreting molecular mechanisms of the TCM formula in treating complex diseases.

## Data Availability

The original contributions presented in the study are included in the Supplementary Material; further inquiries can be directed to the corresponding authors.

## Abbreviations

[Ca^2+^]i, concentration of intracellular Ca^2+^; AC, adenylyl cyclase; AVP, arginine vasopressin; Caco-2, Caco-2 permeability; CIM, core function motifs; C-T, component–target network; CUMS, chronic unpredictable mild stress; DL, drug-likeness; DSS, Danggui-Shaoyao-San; FA, ferulic acid; FCG, functional components group; FST, forced swimming test; GO, Gene Ontology; KEGG, Kyoto Encyclopedia of Genes and Genomes; MAOIs, monoamine oxidase inhibitors; MLogP, Moriguchi octanol–water partition coefficient (LogP); MW, molecular weight; nHAcc, number of acceptor atoms for H-bonds; nHDon, number of donor atoms for H-bonds; OB, Ooral bioavailability; PKA, protein kinase A; PPI, protein–protein interaction network; SEA, similarity ensemble approach; SSRIs, selective serotonin reuptake inhibitors; TCA, target coverage accumulation; TCAs, tricyclic antidepressants; TCM, traditional Chinese medicine; TCMID, Traditional Chinese Medicine Integrated Database; TCMSP, Traditional Chinese Medicine Systems Pharmacology Database; TPSA, topological polar surface area.
