# Supplementary material for: Mathematical algorithm–based identification of the functional components and mechanisms in depression treatment: An example of Danggui-Shaoyao-San
Source: Front Cell Dev Biol. 2022 Aug 22;10:937621. doi: 10.3389/fcell.2022.937621 (PMC9441958; doi:10.3389/fcell.2022.937621)
Supplement: Supplementary file 1 [file DataSheet1.docx]

**Supplementary Material**

**A novel systems pharmacology strategy for uncovering the core functional components and mechanism of Danggui-Shaoyao-San Treatment for Depression**

Wenxia Gong^1, 3, 4, #^, Kexin Wang^2, 5, 6#^, Xueyuan Wang^1^, Yupeng Chen^6^, Xuemei Qin^1, 3, 4,*^, Aiping Lu^5, *^, Daogang Guan^6, 7, *^

^1^ *Modern Research Center for Traditional Chinese Medicine of Shanxi University, Shanxi, China*

*^2^ National Key Clinical Specialty/Engineering Technology Research Center of Education Ministry of China, Guangdong Provincial Key Laboratory on Brain Function Repair and Regeneration, Neurosurgery Institute, Department of Neurosurgery, Guangzhou, China*

^3^ *Key Laboratory of Chemical Biology and Molecular Engineering of Ministry of Education, Shanxi, China*

*^4^ Key Laboratory of Effective Substances Research and Utilization in TCM of Shanxi Province, Shanxi, China*

*^5^ Institute of Integrated Bioinformedicine and Translational Science, Hong Kong Baptist University, Hong Kong, China*

*^6^ Department of Biochemistry and Molecular Biology, School of Basic Medical Sciences, Southern Medical University, Guangzhou, China*

*^7^ Guangdong Key Laboratory of Single Cell Technology and Application, Southern Medical University, Guangzhou, China*

**^*^** **Correspondence authors.** Tel: +86-351-7011202; Fax: +86-351-7011202

1. **mail addresses:** XueMei Qin (qinxm@sxu.edu.cn), DaoGang Guan [(guandg0929@hotmail.com);](mailto:(guandg0929@hotmail.com);) Aiping Lu (aipinglu@hkbu.edu.hk)

**Experimental Section**

**Material and reagents**

Ferulic acid (over 98 % purity by HPLC) was purchased from Chengdu Pufei De Biotech Co., Ltd (Chengdu, China). Fetal bovine serum (FBS), penicillin and streptomycin were purchased from Gibco (Grand Island, USA). RPMI-1640 was purchased from HyClone (GE, USA). 3-(4,5-dimethy thiazol-2-yl)-2,5-diphenyl tetrazolium bromide (MTT) were purchased from Sigma Aldrich (St. Louis, USA). Corticosterone with purity over 98.5% was obtained from TCI (Shanghai, China). All chemicals and reagents are of analytical grade unless stated otherwise.

**Collect Chemical Components of DSS**

All components of DSS were collected from three published natural product data sources: Traditional Chinese Medicine integrated database (TCMID, <http://www.megabionet.org/tcmid/),> Traditional Chinese Medicine Systems Pharmacology Database (TCMSP) (http://lsp.nwsuaf.edu.cn/tcmsp.php), and TCM@Taiwan (http://tcm.cmu.edu.tw/zh-tw). For all components, the initial structure formats (e.g., mol2 and SDF) were transformed into canonical SMILES format using OpenBabel toolkit (version 2.4.1). Subsequently, the properties, such as molecular weight (MW), oral bioavailability (OB), Caco-2 permeability (Caco-2), drug-likeness (DL), Moriguchioctanol-water partition coefficient (LogP) (MLogP), number of acceptor atoms for H-bonds (nHAcc), number of donor atoms for H-bonds (nHDon), and topological polar surface area (TPSA), and GI absorption was retrieved from TCMSP.

**Potential Active Components Selection**

Lipinski’s rule was used to identify druggable compounds according to the following criteria: molecular weight lower than 500 Da (MW ≤500), number of donor hydrogen bonds less than 5 (H-bond donors ≤5), number of donor hydrogen bonds less than 5 (H-bond acceptors ≤10), and the LogP lower than 5 and over -2 (-2＜LogP＜5). Besides, Human intestinal cell line Caco-2 was also employed to screen the active components. The components with Caco-2 ＞-0.4 were retained for further investigation.

**Predict Targets of Active Components**

To obtain the targets of active components in DSS, the commonly used prediction tools, i.e., HitPick, Swiss Target Prediction and Similarity Ensemble Approach (SEA) were used to predict the targets. All chemical structures were prepared and converted into canonical SMILES using OpenBabel toolkit (version 2.4.1). In addition, the targets results were confirmed by literature review.

**Gene Ontology and Pathway Analysis**

To analyze the main function of the targets, the clusterProfiler package of R software was used to perform Gene Ontology (GO) analysis. p-values were set at 0.05 as the cut-off criterion. The clusterProfiler package of R software was employed to classify the biological terms and to analyze the gene cluster enrichment automatically. The latest pathway data were obtained from the Kyoto Encyclopedia of Genes and Genomes (KEGG) database (https://www.kegg.jp/) for KEGG pathway enrichment analyses. p-values were set at 0.05 as the cut-off criterion.

**Cell culturing and treatment**

PC12 cells derived from pheochromocytoma of the rat adrenal medulla were obtained from Institute of Materia Medica, Chinese Academy of Medical Sciences and Peking Union Medical College, Beijing. They were maintained in RPMI medium supplemented with 10 % (v/v) heat-inactivated FBS, 100 U/mL penicillin and 100 mg/mL streptomycin, at 37 °C and 5% CO_2_ under a humidiﬁed atmosphere. For all experiments, cells in the exponential phase of growth were used.

Desipramine, vanillic acid, anisic acid, ferulic acid was hemolyzeed in dimethyl sulphoxide (DMSO). The final concentration of DMSO was not more than 0.1% (v/v). For cell cytotoxicity assays, PC12 cells were incubated at a density of 2×10^4^ cells per well in 96-well microplates and then maintained for a 28h incubation with test compounds. For neuroprotective assay, PC12 cells were treated with test compounds for 4 h before exposure to 400 μM corticosterone and then maintained for 24 h.

**MTT assay**

The effects of the test compounds on the cytotoxicity were determined by the MTT assay as described previously. Briefly, PC12 cells were seeded in 96-well plates at 2×10^4^ cells per well and co-incubated with the test compounds for 24 h. Cultures were also treated with 0.1% DMSO as the untreated control. After treatment, 10μL of MTT solution (5 mg/mL) was added to each well and the plates were incubated for 4 h at 37°C. The supernatant was then removed from formazan crystals and 100μL of DMSO was added to each well. The absorbance at 570 nm was recorded usinga microplate reader (BioTek, USA).The cell viability was expressed as a percentage of the absorbance value determined for the control cultures.

**Apoptosis assay**

To further investigate the cytoprotective effects of ferulic acid, Hoechst 33342 and PI double fluorescent staining were assayed. The PC12 cells (4 × 10^5^ per well) were seeded in a 6-well plate. At the end of the drug treatment, the cells were incubated with 5 mg.mL^-1^ Hoechst 33342 for 10 min, washed twice with PBS, incubated with 1 μg.mL^-1^ PI working solution for an additional 10 min, and then visualized using inverted fluorescence microscopy (IX71, Olympus, Japan).

**Measurement of intracellular calcium level ([Ca^2+^]i)**

The concentration of intracellular Ca^2+^([Ca^2+^]i) was measured with Fluo-4/AM. At the end of treatment, PC12 cells were collected and centrifuged to get the supernatant, then incubatedwith Fluo-4/AM (5 μmol/L) at 37 °C for 1 h, and then centrifuged twice at 1000 rpm for 5 min. The cells were re-suspended in D-hanks buffer solution. At the end of the drug treatment, the cells were stained in a dish and then visualized using inverted fluorescence microscopy.

**Animals and administrations**

Male ICR mice (18-22 g) were purchased from the Beijing Vital Laboratory Animal Co. Ltd (Beijing, China). The mice were maintained under a 12 h light-12 h dark cycle and free access to water or food throughout the study. All experimental procedures in this study were performed in accordance with the NIH Guide for the Care and Use of Laboratory Animals.

Venlafaxine (50 mg/kg) served as positive control group. The doses of the treated groups were designed as 25, 50 and 100 mg/kg of ferulic acid. The solutions of the tested samples were administered to the mice via gastric intubation at a dosage of 0.2 mL/10 g (body weight) once daily between 9:00 a.m. to 10:00 a.m for 14 days. Behavioral tests were carried as follows: tail suspension test (13^th^ day) and forced-swimming test (14^th^ day).

**Tail suspension test**

The TST was carried out according to the method of Steru et al (1985). The mouse was hung by the tail (clipped 2 cm from the end) for 6 min in a box of dimensions 50 cm × 25 cm × 50 cm, its head 15 cm above the bottom of the box. Data was recorded only in the ﬁnal 4 min of the test. Immobility was defined as the absence of any limb or body movements. With the exception of those caused by respiration, when the mice hang passively and completely motionless. Animals that climbed their tails during testing were excluded from the analysis. During the test session, the immobility time was recorded using a video camera. Two observers who had no knowledge of the type of treatment each animal had received evaluated the tapes.

**Forced swim test**

The FST was carried out on mice according to the method of Porsolt (1977). Briefly, mice were individually placed into a glass cylinder (20 cm in height, 14 cm in diameter) filled with 12 cm high water (25 ± 1˚C). The total duration of immobility (seconds) was measured during the last 4 min of a single 6 min test session. Mice were considered immobile when they made no attempts to escape except for the movements necessary to keep their heads above the water. The definition of immobility was the absence of all movements with the exception of motions required to maintain the animal’s head above the water. The results are expressed as the time spent immobile during the last 4 min of the 6 min session. After that, the mice were sacrificed through the dislocation of cervical vertebra.

**Statistical analysis**

Data were expressed as the mean ± SEM. Multiple group comparisons were performed with one-way ANOVA to compare the control and treatment groups; *P*< 0.05 were considered as statistically significant. All statistical analyses were performed with the SPSS 19.0 software. All experiments were performed a minimum of three times.

**Result Section**


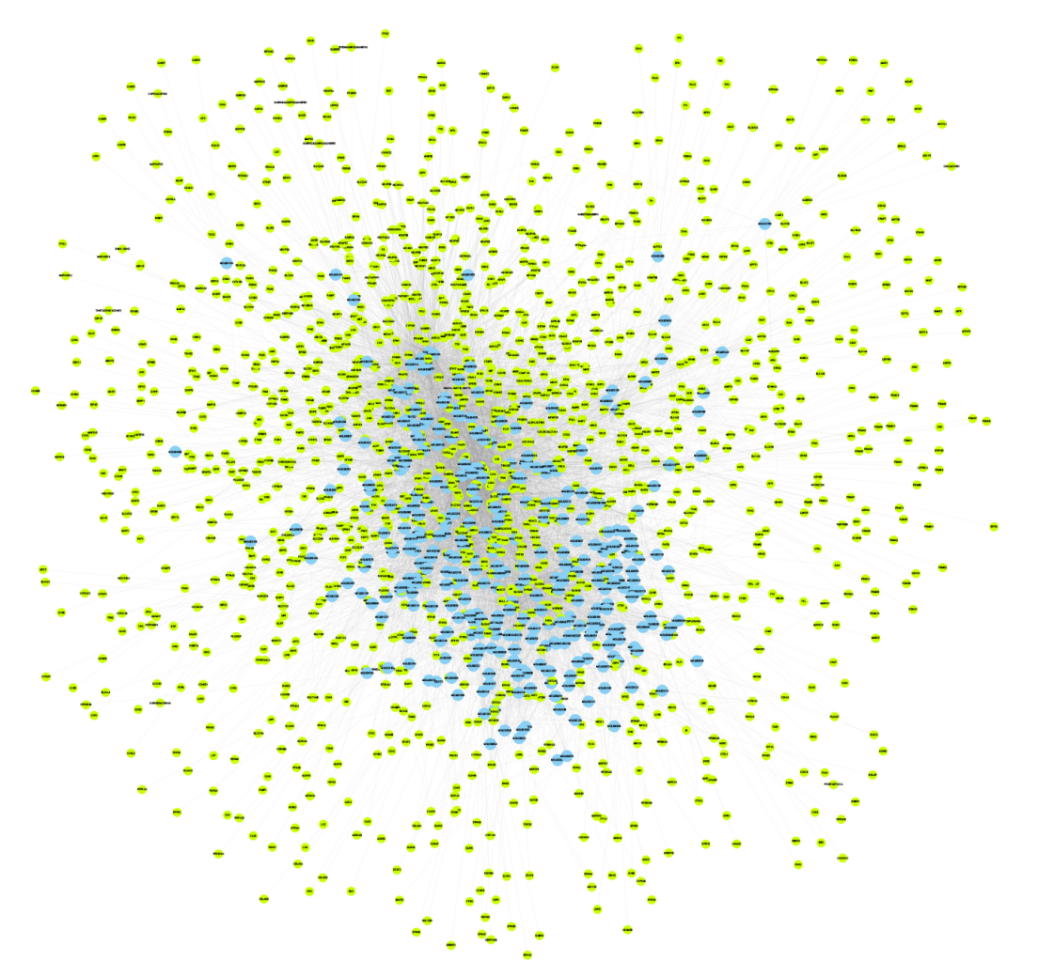


**Figure S1.** The component-target network.

**Table S1.** Components in DSS for further analysis after ADME screening.

| ID | Component | MW | LogP | Caco-2 | HDON | HACC | RBN | Source |
| --- | --- | --- | --- | --- | --- | --- | --- | --- |
| DSS1 | Hemo-sol | 136.26 | 3.47 | 1.83 | 0 | 0 | 1 | Danggui |
| DSS2 | β-Selinene | 204.39 | 4.52 | 1.83 | 0 | 0 | 1 | Danggui |
| DSS3 | Scopoletol | 192.18 | 1.42 | 0.71 | 1 | 4 | 1 | Danggui |
| DSS4 | Nonanal | 142.27 | 2.76 | 1.31 | 0 | 1 | 7 | Danggui |
| DSS5 | Cymol | 134.24 | 3.55 | 1.86 | 0 | 0 | 1 | Danggui |
| DSS6 | Decanal | 156.30 | 3.14 | 1.34 | 0 | 1 | 8 | Danggui |
| DSS7 | (-)-α-Pinene | 136.26 | 3.60 | 1.85 | 0 | 0 | 0 | Danggui |
| DSS8 | β-Chamigrene | 204.39 | 4.34 | 1.82 | 0 | 0 | 0 | Danggui |
| DSS9 | Myrcene | 136.26 | 3.53 | 1.84 | 0 | 0 | 4 | Danggui |
| DSS10 | Safrol | 162.20 | 2.40 | 1.44 | 0 | 2 | 2 | Danggui |
| DSS11 | p-Ocimene | 136.26 | 3.53 | 1.85 | 0 | 0 | 3 | Danggui |
| DSS12 | Moslene | 136.26 | 3.45 | 1.88 | 0 | 0 | 1 | Danggui |
| DSS13 | isoeugenol | 164.22 | 2.44 | 1.38 | 1 | 2 | 2 | Danggui |
| DSS14 | ()-α-Terpineol | 154.28 | 2.68 | 1.28 | 1 | 1 | 1 | Danggui |
| DSS15 | o-Thymol | 150.24 | 2.83 | 1.58 | 1 | 1 | 1 | Danggui |
| DSS16 | CHEBI:7 | 136.26 | 3.58 | 1.84 | 0 | 0 | 0 | Danggui |
| DSS17 | Ferulic acid | 194.20 | 1.38 | 0.47 | 2 | 4 | 3 | Danggui |
| DSS18 | Ferulic acid (CIS) | 194.20 | 1.38 | 0.53 | 2 | 4 | 3 | Danggui |
| DSS19 | nicotinic acid | 123.12 | 0.22 | 0.34 | 1 | 3 | 1 | Danggui |
| DSS20 | Eucarvone | 150.24 | 2.34 | 1.35 | 0 | 1 | 0 | Danggui |
| DSS21 | Farnesene | 204.39 | 4.98 | 1.95 | 0 | 0 | 7 | Danggui |
| DSS22 | (1S,4aR,8aR)-1-isopropyl-7-methyl-4-methylene-2,3,4a,5,6,8a-hexahydro-1H-naphthalene | 204.39 | 4.23 | 1.86 | 0 | 0 | 1 | Danggui |
| DSS23 | vanillin | 152.16 | 1.13 | 0.68 | 1 | 3 | 2 | Danggui |
| DSS24 | WLN: VH6 | 114.21 | 2.06 | 1.29 | 0 | 1 | 5 | Danggui |
| DSS25 | bergamotene | 204.39 | 4.72 | 1.86 | 0 | 0 | 3 | Danggui |
| DSS26 | cuminal | 148.22 | 2.36 | 1.39 | 0 | 1 | 2 | Danggui |
| DSS27 | 80-57-9 | 150.24 | 2.20 | 1.27 | 0 | 1 | 0 | Danggui |
| DSS28 | Usaf hc-1 | 202.28 | 1.93 | -0.01 | 2 | 4 | 9 | Danggui |
| DSS29 | o-Acetyl-p-cresol | 150.19 | 1.90 | 1.02 | 1 | 2 | 1 | Danggui |
| DSS30 | Azelex | 188.25 | 1.54 | -0.04 | 2 | 4 | 8 | Danggui |
| DSS31 | (+)-Ledol | 222.41 | 3.53 | 1.43 | 1 | 1 | 0 | Danggui |
| DSS32 | Hypnon | 120.16 | 1.72 | 1.36 | 0 | 1 | 1 | Danggui |
| DSS33 | NON | 172.30 | 3.10 | 0.96 | 1 | 2 | 8 | Danggui |
| DSS34 | adenine | 135.15 | -0.19 | -0.30 | 3 | 4 | 0 | Danggui |
| DSS35 | ()-Cuparene | 202.37 | 4.63 | 1.88 | 0 | 0 | 1 | Danggui |
| DSS36 | cis-Thujopsene | 204.39 | 4.53 | 1.84 | 0 | 0 | 0 | Danggui |
| DSS37 | 3-Butylidene-7-hydroxyphthalide | 204.24 | 2.65 | 1.00 | 1 | 3 | 2 | Danggui |
| DSS38 | Levistolide A | 380.52 | 4.55 | 0.94 | 0 | 4 | 4 | Danggui |
| DSS39 | Allocymene | 136.26 | 3.54 | 1.85 | 0 | 0 | 2 | Danggui |
| DSS40 | BdPh | 188.24 | 2.83 | 1.32 | 0 | 2 | 2 | Danggui |
| DSS41 | senkyunolide-C | 204.24 | 2.41 | 0.87 | 1 | 3 | 2 | Danggui |
| DSS42 | senkyunolide-D | 222.26 | 1.27 | 0.12 | 1 | 4 | 3 | Danggui |
| DSS43 | senkyunolide-E | 204.24 | 1.99 | 0.55 | 1 | 3 | 2 | Danggui |
| DSS44 | 4-Octanone | 128.24 | 2.35 | 1.37 | 0 | 1 | 5 | Danggui |
| DSS45 | (6R)-6-butylcyclohepta-1,4-diene | 150.29 | 3.65 | 1.85 | 0 | 0 | 3 | Danggui |
| DSS46 | cis-ligustilide | 190.26 | 2.68 | 1.30 | 0 | 2 | 2 | Danggui |
| DSS47 | Methylbutenol | 86.15 | 1.12 | 1.12 | 1 | 1 | 1 | Danggui |
| DSS48 | PCR | 108.15 | 1.73 | 1.56 | 1 | 1 | 0 | Danggui |
| DSS49 | (4S)-1-methyl-4-(6-methylhepta-1,5-dien-2-yl)cyclohexene | 204.39 | 4.92 | 1.89 | 0 | 0 | 4 | Danggui |
| DSS50 | Guasol | 124.15 | 1.41 | 1.28 | 1 | 2 | 1 | Danggui |
| DSS51 | IPH | 94.12 | 1.39 | 1.50 | 1 | 1 | 0 | Danggui |
| DSS52 | CADINENE | 204.39 | 4.14 | 1.88 | 0 | 0 | 1 | Danggui |
| DSS53 | Acoradiene | 204.39 | 4.44 | 1.85 | 0 | 0 | 1 | Danggui |
| DSS54 | Maruzen M | 122.18 | 2.10 | 1.57 | 1 | 1 | 1 | Danggui |
| DSS55 | o-cresol | 108.15 | 1.75 | 1.57 | 1 | 1 | 0 | Danggui |
| DSS56 | 6,7,3',8'-diligustilide | 380.52 | 4.41 | 0.79 | 0 | 4 | 4 | Danggui |
| DSS57 | β-Terpinene | 136.26 | 3.01 | 1.85 | 0 | 0 | 1 | Danggui |
| DSS58 | Butal | 72.12 | 0.94 | 1.18 | 0 | 1 | 2 | Danggui |
| DSS59 | Anisic acid | 152.16 | 1.56 | 0.69 | 1 | 3 | 2 | Danggui |
| DSS60 | h-Met-h | 149.24 | -0.70 | 0.06 | 3 | 3 | 4 | Danggui |
| DSS61 | 3,4-dimethylbenzaldehyde | 134.19 | 2.01 | 1.40 | 0 | 1 | 1 | Danggui |
| DSS62 | Dimethylbenzaldehyde | 134.19 | 2.04 | 1.40 | 0 | 1 | 2 | Danggui |
| DSS63 | TMHYDROP | 152.21 | 1.84 | 1.21 | 2 | 2 | 0 | Danggui |
| DSS64 | bicycloelemene | 204.39 | 4.47 | 1.88 | 0 | 0 | 0 | Danggui |
| DSS65 | WLN: QR CQ DV1 | 152.16 | 1.21 | 0.67 | 2 | 3 | 1 | Danggui |
| DSS66 | WLN: QVR BVQ | 166.14 | 0.90 | -0.05 | 2 | 4 | 2 | Danggui |
| DSS67 | ESEN | 148.12 | 1.35 | 0.56 | 0 | 3 | 0 | Danggui |
| DSS68 | sedanolide | 194.30 | 2.86 | 1.24 | 0 | 2 | 3 | Danggui |
| DSS69 | senkyunolide | 192.28 | 2.67 | 1.28 | 0 | 2 | 3 | Danggui |
| DSS70 | α-acoradiene | 204.39 | 4.37 | 1.82 | 0 | 0 | 1 | Danggui |
| DSS71 | InChI=1/C15H24/c1-10-7-8-15-9-12(10)14(3,4)13(15)6-5-11(15)2/h7,11-13H,5-6,8-9H2,1-4H | 204.39 | 4.47 | 1.79 | 0 | 0 | 0 | Danggui |
| DSS72 | α-copaene | 204.39 | 4.43 | 1.83 | 0 | 0 | 1 | Danggui |
| DSS73 | (1R,4R,5S)-4-isopropenyl-1,8-dimethylspiro[4.5]dec-8-ene | 204.39 | 4.43 | 1.83 | 0 | 0 | 1 | Danggui |
| DSS74 | 2,6-di(phenyl)thiopyran-4-thione | 280.43 | 4.49 | 1.74 | 0 | 0 | 2 | Danggui |
| DSS75 | o-Xylenol | 122.18 | 2.11 | 1.62 | 1 | 1 | 0 | Danggui |
| DSS76 | 2,4,6-trimethyl-Octane | 156.35 | 4.45 | 1.81 | 0 | 0 | 5 | Danggui |
| DSS77 | Mesitaldehyde | 148.22 | 2.35 | 1.54 | 0 | 1 | 1 | Danggui |
| DSS78 | Isoxylaldehyde | 134.19 | 2.05 | 1.39 | 0 | 1 | 1 | Danggui |
| DSS79 | 2-valerylbenzoic acid | 206.26 | 2.24 | 0.61 | 1 | 3 | 5 | Danggui |
| DSS80 | (Z)-2-Hexenyl hexanoate | 198.34 | 3.40 | 1.26 | 0 | 2 | 9 | Danggui |
| DSS81 | cis-Isoeugenol | 164.22 | 2.44 | 1.43 | 1 | 2 | 2 | Danggui |
| DSS82 | 2-methyldodecan-5-one | 198.39 | 4.02 | 1.43 | 0 | 1 | 9 | Danggui |
| DSS83 | 3,7-dimethyl-nonane | 156.35 | 4.58 | 1.78 | 0 | 0 | 6 | Danggui |
| DSS84 | 1,1,5-trimethyl-2-formylcyclohexa-2,5-diene-4-one | 164.22 | 1.50 | 0.82 | 0 | 2 | 1 | Danggui |
| DSS85 | 4-Methyl-6-hepten-3-one | 126.22 | 2.08 | 1.39 | 0 | 1 | 4 | Danggui |
| DSS86 | 6-Ethylresorcinol | 138.18 | 1.49 | 1.13 | 2 | 2 | 1 | Danggui |
| DSS87 | 5-Indolol | 133.16 | 1.24 | 1.38 | 2 | 1 | 0 | Danggui |
| DSS88 | Undecanol-6 | 172.35 | 3.55 | 1.19 | 1 | 1 | 8 | Danggui |
| DSS89 | 7,10-PENTADECADIYNOIC ACID | 234.37 | 3.85 | 1.32 | 1 | 2 | 7 | Danggui |
| DSS90 | Amyl ketone | 170.33 | 3.44 | 1.31 | 0 | 1 | 8 | Danggui |
| DSS91 | Isoamylbenzene | 148.27 | 3.91 | 1.84 | 0 | 0 | 3 | Danggui |
| DSS92 | (Z)-2-[[(Z)-2-methylbut-2-enoyl]oxymethyl]but-2-enoic acid | 198.24 | 1.61 | 0.49 | 1 | 4 | 5 | Danggui |
| DSS93 | Tropone | 106.13 | 0.95 | 1.25 | 0 | 1 | 0 | Danggui |
| DSS94 | aromadendrene | 204.39 | 4.48 | 1.83 | 0 | 0 | 0 | Danggui |
| DSS95 | BUA | 88.12 | 0.81 | 0.69 | 1 | 2 | 2 | Danggui |
| DSS96 | (3S)-3-butyl-3H-isobenzofuran-1-one | 190.26 | 2.69 | 1.30 | 0 | 2 | 3 | Danggui |
| DSS97 | ()-Camphoric acid | 200.26 | 1.27 | 0.10 | 2 | 4 | 2 | Danggui |
| DSS98 | (3E)-3-butylidene-7-hydroxy-2-benzofuran-1-one | 204.24 | 2.63 | 1.03 | 1 | 3 | 2 |  |
| DSS99 | Coniferyl ferulate | 356.40 | 3.16 | 0.71 | 2 | 6 | 8 | Danggui |
| DSS100 | 2,4-Xylylaldehyde | 134.19 | 2.18 | 1.42 | 0 | 1 | 1 | Danggui |
| DSS101 | m-Ethylphenol | 122.18 | 2.05 | 1.55 | 1 | 1 | 1 | Danggui |
| DSS102 | 1,5,5,6-tetramethyl-1,3-Cyclohexadiene | 136.26 | 3.08 | 1.82 | 0 | 0 | 0 | Danggui |
| DSS103 | PYG | 126.12 | 0.62 | 0.69 | 3 | 3 | 0 | Baishao |
| DSS104 | BOX | 121.12 | 1.49 | 0.54 | 0 | 2 | 1 | Baishao |
| DSS105 | kaempferol | 286.25 | 1.46 | 0.26 | 4 | 6 | 1 | Baishao |
| DSS106 | 3,4,5-trihydroxybenzoic acid | 170.13 | 0.31 | -0.09 | 4 | 5 | 1 | Baishao |
| DSS107 | (-)-α-cedrene | 204.39 | 4.47 | 1.81 | 0 | 0 | 0 | Baishao |
| DSS108 | EEE | 88.12 | 0.87 | 1.07 | 0 | 2 | 2 | Baishao |
| DSS109 | paeonol | 166.19 | 1.62 | 0.93 | 1 | 3 | 2 | Baishao |
| DSS110 | Cedrol | 222.41 | 3.57 | 1.35 | 1 | 1 | 0 | Baishao |
| DSS111 | (1R)-()-Nopinone | 138.23 | 1.91 | 1.23 | 0 | 1 | 0 | Baishao |
| DSS112 | salicylic acid | 138.13 | 1.37 | 0.63 | 2 | 3 | 1 | Baishao |
| DSS113 | 2,2-dimethylcyclohexanol | 128.24 | 2.02 | 1.22 | 1 | 1 | 0 | Baishao |
| DSS114 | 9-methylenefluorene | 178.24 | 3.68 | 1.95 | 0 | 0 | 0 | Baishao |
| DSS115 | Dipropyl phthalate | 250.32 | 3.03 | 0.78 | 0 | 4 | 8 | Baishao |
| DSS116 | BU3 | 90.14 | -0.05 | 0.19 | 2 | 2 | 1 | Baishao |
| DSS117 | Dibutylphenol | 206.36 | 3.96 | 1.73 | 1 | 1 | 2 | Baishao |
| DSS118 | bicyclo[3.1.1]hept-2-ene-2-methanol, 6,6-dimethyl- | 152.26 | 2.48 | 1.23 | 1 | 1 | 1 | Baishao |
| DSS119 | ()-trans-Myrtanol | 154.28 | 2.57 | 1.17 | 1 | 1 | 1 | Baishao |
| DSS120 | Acetyl oxide | 102.1 | 0.39 | 0.65 | 0 | 3 | 2 | Baishao |
| DSS121 | 2-methyl-3-(2-propenyl)-phenol | 148.22 | 2.57 | 1.64 | 1 | 1 | 2 | Baishao |
| DSS122 | Methylgallate | 184.16 | 0.65 | 0.26 | 3 | 5 | 2 | Baishao |
| DSS123 | Progallin A | 198.19 | 1.00 | 0.33 | 3 | 5 | 3 | Baishao |
| DSS124 | (Z)-(1S,5R)-β-pinen-10-yl-β-vicianoside | 152.26 | 2.76 | 1.52 | 1 | 1 | 0 | Baishao |
| DSS125 | 11α,12α-epoxy-3β-23-dihydroxy-30-norolean-20-en-28,12β-olide | 470.71 | 4.40 | 0.09 | 2 | 5 | 1 | Baishao |
| DSS126 | propyl (2R)-2-hydroxypropanoate | 132.18 | 0.84 | 0.44 | 1 | 3 | 4 | Baishao |
| DSS127 | (3S,3aR,5S,6S,7aR)-5,6-dihydroxy-3,6-dimethyl-3,3a,4,5,7,7a-hexahydrobenzofuran-2-one | 200.26 | 0.46 | -0.16 | 2 | 4 | 0 | Baishao |
| DSS128 | paeoniflorgenone | 318.35 | 1.44 | -0.09 | 1 | 6 | 4 | Baishao |
| DSS129 | (3S,5R,8R,9R,10S,14S)-3,17-dihydroxy-4,4,8,10,14-pentamethyl-2,3,5,6,7,9-hexahydro-1H-cyclopenta[a]phenanthrene-15,16-dione | 358.52 | 3.08 | 0.00 | 2 | 4 | 0 | Baishao |
| DSS130 | [(3S,3aR,6S,7aR)-6-hydroxy-6-methyl-2,5-dioxo-3a,4,7,7a-tetrahydro-3H-benzofuran-3-yl]methyl benzoate | 318.35 | 1.41 | -0.17 | 1 | 6 | 4 | Baishao |
| DSS131 | paeoniflorin | 318.35 | 1.27 | -0.34 | 2 | 6 | 4 | Baishao |
| DSS132 | 4-Chlorobutyric acid | 122.56 | 0.93 | 0.74 | 1 | 2 | 2 | Baishao |
| DSS133 | (3R,3aR,6S,7aR)-6-hydroxy-3,6-dimethyl-3a,4,7,7a-tetrahydro-3H-benzofuran-2,5-dione | 198.24 | 0.49 | -0.08 | 1 | 4 | 0 | Baishao |
| DSS134 | (3aR,6S,7aR)-6-hydroxy-6-methyl-3-methylene-3a,4,7,7a-tetrahydrobenzofuran-2,5-dione | 196.22 | 0.39 | -0.01 | 1 | 4 | 0 | Baishao |
| DSS135 | cis-5-Octen-1-ol | 128.24 | 2.17 | 1.16 | 1 | 1 | 5 | Baishao |
| DSS136 | 24253-30-3 | 98.16 | 1.45 | 1.33 | 0 | 1 | 3 | Baishao |
| DSS137 | acetic acid | 60.06 | 0.01 | 0.42 | 1 | 2 |  | Baishao |
| DSS138 | Ethylisobutyrate | 116.18 | 1.54 | 1.24 | 0 | 2 |  | Baishao |
| DSS139 | D-Camphene | 136.26 | 3.52 | 1.81 | 0 | 0 | 0 | Chuanxiong |
| DSS140 | α-humulene | 204.39 | 4.35 | 1.88 | 0 | 0 | 0 | Chuanxiong |
| DSS141 | α-Curcumene | 202.37 | 4.84 | 1.93 | 0 | 0 | 4 | Chuanxiong |
| DSS142 | 2-[(1R,3S,4S)-3-isopropenyl-4-methyl-4-vinylcyclohexyl]propan-2-ol | 222.41 | 3.78 | 1.37 | 1 | 1 | 3 | Chuanxiong |
| DSS143 | PHB | 138.13 | 1.13 | 0.39 | 2 | 3 | 1 | Chuanxiong |
| DSS144 | vanillic acid | 168.16 | 1.17 | 0.43 | 2 | 4 | 2 | Chuanxiong |
| DSS145 | (L)-α-Terpineol | 154.28 | 2.57 | 1.39 | 1 | 1 | 1 | Chuanxiong |
| DSS146 | 1,8-cineole | 154.28 | 2.63 | 1.57 | 0 | 1 | 0 | Chuanxiong |
| DSS147 | (-)-nopinene | 136.26 | 3.51 | 1.80 | 0 | 0 | 0 | Chuanxiong |
| DSS148 | 2-[(2S,5S,6S)-6,10-dimethylspiro[4.5]dec-9-en-2-yl]propan-2-ol | 222.41 | 3.59 | 1.44 | 1 | 1 | 1 | Chuanxiong |
| DSS149 | Furol | 96.09 | 0.49 | 1.08 | 0 | 2 | 1 | Chuanxiong |
| DSS150 | L-Bornyl acetate | 196.32 | 3.09 | 1.29 | 0 | 2 | 2 | Chuanxiong |
| DSS151 | (R)-linalool | 154.28 | 2.74 | 1.33 | 1 | 1 | 4 | Chuanxiong |
| DSS152 | (S)-(+)-α-Phellandrene | 136.26 | 3.07 | 1.87 | 0 | 0 | 1 | Chuanxiong |
| DSS153 | cis-β.-Elemene diastereomer | 204.39 | 4.70 | 1.85 | 0 | 0 | 3 | Chuanxiong |
| DSS154 | Methyleugenol | 178.25 | 2.48 | 1.47 | 0 | 2 | 4 | Chuanxiong |
| DSS155 | ()-Aromadendrene | 204.39 | 4.47 | 1.81 | 0 | 0 | 0 | Chuanxiong |
| DSS156 | caffeic acid | 180.17 | 0.98 | 0.21 | 3 | 4 | 2 | Chuanxiong |
| DSS157 | L-Limonen | 136.26 | 3.45 | 1.83 | 0 | 0 | 1 | Chuanxiong |
| DSS158 | Tereben | 136.26 | 3.48 | 1.86 | 0 | 0 | 0 | Chuanxiong |
| DSS159 | (1S,5S)-1-isopropyl-4-methylenebicyclo[3.1.0]hexane | 136.26 | 3.26 | 1.83 | 0 | 0 | 1 | Chuanxiong |
| DSS160 | Undekansaeure | 186.33 | 3.41 | 0.98 | 1 | 2 | 9 | Chuanxiong |
| DSS161 | ()-Terpinen-4-ol | 154.28 | 2.66 | 1.36 | 1 | 1 | 1 | Chuanxiong |
| DSS162 | hexanal | 100.18 | 1.68 | 1.25 | 0 | 1 | 4 | Chuanxiong |
| DSS163 | PENTYLFURAN | 138.23 | 2.79 | 1.72 | 0 | 1 | 4 | Chuanxiong |
| DSS164 | (R)-(-)-α-Phellandrene | 136.26 | 3.07 | 1.86 | 0 | 0 | 1 | Chuanxiong |
| DSS165 | OYA | 128.24 | 2.41 | 1.30 | 0 | 1 | 6 | Chuanxiong |
| DSS166 | o-Cymol | 134.24 | 3.60 | 1.88 | 0 | 0 | 1 | Chuanxiong |
| DSS167 | β-elemene | 204.39 | 4.69 | 1.84 | 0 | 0 | 3 | Chuanxiong |
| DSS168 | Terpilene | 136.26 | 3.38 | 1.84 | 0 | 0 | 1 | Chuanxiong |
| DSS169 | (R)-p-Menth-1-en-4-ol | 154.28 | 2.64 | 1.33 | 1 | 1 | 1 | Chuanxiong |
| DSS170 | gamma.-Bisabolene | 204.39 | 4.90 | 1.94 | 0 | 0 | 3 | Chuanxiong |
| DSS171 | 58870_FLUKA | 204.39 | 4.37 | 1.82 | 0 | 0 | 0 | Chuanxiong |
| DSS172 | 5-isopropyl-2-methylbicyclo[3.1.0]hex-2-ene | 136.26 | 3.20 | 1.82 | 0 | 0 | 1 | Chuanxiong |
| DSS173 | (1R,5R,7S)-4,7-dimethyl-7-(4-methylpent-3-enyl)bicyclo[3.1.1]hept-3-ene | 204.39 | 4.84 | 1.86 | 0 | 0 | 3 | Chuanxiong |
| DSS174 | (4S)-4-isopropylcyclohexene-1-carbaldehyde | 152.26 | 2.42 | 1.36 | 0 | 1 | 2 | Chuanxiong |
| DSS175 | (S)-2,2,3-Trimethylcyclopent-3-ene-1-acetaldehyde | 152.26 | 2.19 | 1.32 | 0 | 1 | 2 | Chuanxiong |
| DSS176 | WLN: Q1R | 108.15 | 1.33 | 1.08 | 1 | 1 | 1 | Chuanxiong |
| DSS177 | 49070_FLUKA | 222.41 | 3.51 | 1.29 | 1 | 1 | 0 | Chuanxiong |
| DSS178 | Crysophanol | 254.25 | 2.21 | 0.62 | 2 | 4 | 0 | Chuanxiong |
| DSS179 | uracil | 112.1 | -0.57 | 0.05 | 2 | 4 | 0 | Chuanxiong |
| DSS180 | EUG | 150.19 | 2.15 | 1.36 | 1 | 2 | 2 | Chuanxiong |
| DSS181 | (+)-β-Phellandrene | 136.26 | 3.13 | 1.83 | 0 | 0 | 1 | Chuanxiong |
| DSS182 | thymol | 150.24 | 2.80 | 1.60 | 1 | 1 | 1 | Chuanxiong |
| DSS183 | α-Cubebene | 204.39 | 4.45 | 1.83 | 0 | 0 | 1 | Chuanxiong |
| DSS184 | (+)-α-funebrene | 204.39 | 4.48 | 1.79 | 0 | 0 | 0 | Chuanxiong |
| DSS185 | gem-Dimethylcyclopentane | 98.21 | 2.93 | 1.78 | 0 | 0 | 0 | Chuanxiong |
| DSS186 | Senkyunolide-K | 208.28 | 1.81 | 0.52 | 1 | 3 | 3 | Chuanxiong |
| DSS187 | (3Z,6S,7R)-3-butylidene-6-butyryl-7-hydroxy-4,5,6,7-tetrahydroisobenzofuran-1-one | 278.38 | 2.42 | 0.08 | 1 | 4 | 5 | Chuanxiong |
| DSS188 | Senkyunolide-N | 226.3 | 1.03 | -0.15 | 2 | 4 | 3 | Chuanxiong |
| DSS189 | Senkyunolide-P | 382.54 | 4.62 | 0.92 | 0 | 4 | 5 | Chuanxiong |
| DSS190 | Senkyunolide-Q | 278.38 | 2.32 | 0.42 | 1 | 4 | 5 | Chuanxiong |
| DSS191 | 1,1-Diethoxybutane | 146.26 | 2.19 | 1.30 | 0 | 2 | 6 | Chuanxiong |
| DSS192 | Valerophenone | 162.25 | 2.86 | 1.46 | 0 | 1 | 4 | Chuanxiong |
| DSS193 | (1S,5S)-7,7-dimethyl-2-methylenebicyclo[3.1.1]hept-3-ene | 134.24 | 2.86 | 1.80 | 0 | 0 | 0 | Chuanxiong |
| DSS194 | Z-6,8',7,3'-diligustilide | 380.52 | 4.42 | 0.74 | 0 | 4 | 4 | Chuanxiong |
| DSS195 | α-Selinene | 218.42 | 4.78 | 1.82 | 0 | 0 | 1 | Chuanxiong |
| DSS196 | 1,2,3,4,4a,7-Hexahydro-l,6-dimethyl-4-(1-methylethyl)-naphthalene | 162.3 | 3.47 | 1.88 | 0 | 0 | 0 | Chuanxiong |
| DSS197 | m-Ethyltoluene | 120.21 | 3.26 | 1.87 | 0 | 0 | 1 | Chuanxiong |
| DSS198 | (1R,4S,5R)-4-isopropenyl-1,8-dimethylspiro[4.5]dec-8-ene | 204.39 | 4.45 | 1.85 | 0 | 0 | 1 | Chuanxiong |
| DSS199 | β-sesquiphellandrene | 204.39 | 4.62 | 1.89 | 0 | 0 | 4 | Chuanxiong |
| DSS200 | (4aS,7S,8aR)-7-isopropenyl-4a-methyl-1-methylenedecalin | 204.39 | 4.52 | 1.83 | 0 | 0 | 1 | Chuanxiong |
| DSS201 | Artemisia triene | 136.26 | 3.50 | 1.84 | 0 | 0 | 3 | Chuanxiong |
| DSS202 | betea-CUBEBENE | 204.39 | 4.51 | 1.82 | 0 | 0 | 1 | Chuanxiong |
| DSS203 | (1S,4E,8E,10R)-4,8,11,11-tetramethylbicyclo[8.1.0]undeca-4,8-diene | 204.39 | 4.21 | 1.86 | 0 | 0 | 0 | Chuanxiong |
| DSS204 | (Z)-Ligustilide | 188.24 | 2.84 | 1.30 | 0 | 2 | 2 | Chuanxiong |
| DSS205 | Chuanxiongol | 218.27 | 2.34 | 0.94 | 1 | 3 | 2 | Chuanxiong |
| DSS206 | β-asarone | 208.28 | 2.66 | 1.45 | 0 | 3 | 4 | Chuanxiong |
| DSS207 | cis-Piperitol | 154.28 | 2.34 | 1.27 | 1 | 1 | 1 | Chuanxiong |
| DSS208 | (1S,4R,5R)-1-isopropyl-4-methyl-4-bicyclo[3.1.0]hexanol | 154.28 | 2.31 | 1.17 | 1 | 1 | 1 | Chuanxiong |
| DSS209 | Cnidilide | 194.3 | 2.77 | 1.21 | 0 | 2 | 3 | Chuanxiong |
| DSS210 | 1,3,8-p-Menthatriene | 134.24 | 3.00 | 1.86 | 0 | 0 | 1 | Chuanxiong |
| DSS211 | CYCLODODECENE | 166.34 | 4.50 | 1.84 | 0 | 0 | 0 | Chuanxiong |
| DSS212 | cyclohexane,1,1,2,3-tetramethyl- | 140.3 | 3.76 | 1.77 | 0 | 0 | 0 | Chuanxiong |
| DSS213 | 2,9-Dimethyldecane | 170.38 | 4.97 | 1.82 | 0 | 0 | 7 | Chuanxiong |
| DSS214 | (2R,4aR)-2-isopropenyl-4a,8-dimethyl-2,3,4,5,6,7-hexahydro-1H-naphthalene | 204.39 | 4.44 | 1.86 | 0 | 0 | 1 | Chuanxiong |
| DSS215 | 1,5,5-trimethyl-6-methylenecyclohexene | 136.26 | 3.06 | 1.83 | 0 | 0 | 0 | Chuanxiong |
| DSS216 | Isobutyrophenone | 148.22 | 2.45 | 1.46 | 0 | 1 | 2 | Chuanxiong |
| DSS217 | Myricanone | 356.45 | 3.17 | 0.67 | 2 | 5 | 2 | Chuanxiong |
| DSS218 | neocnidilide | 194.3 | 2.87 | 1.23 | 0 | 2 | 3 | Chuanxiong |
| DSS219 | OCT | 114.26 | 3.91 | 1.78 | 0 | 0 | 5 | Chuanxiong |
| DSS220 | p-Cymen-8-ol | 150.24 | 2.19 | 1.33 | 1 | 1 | 1 | Chuanxiong |
| DSS221 | APH | 150.2 | 1.16 | 1.17 | 2 | 3 | 2 | Chuanxiong |
| DSS222 | Perlolyrine | 264.3 | 2.23 | 0.88 | 2 | 3 | 2 | Chuanxiong |
| DSS223 | PLO | 316.53 | 3.99 | 0.69 | 1 | 2 | 1 | Chuanxiong |
| DSS224 | sedanoic-acid | 210.3 | 2.16 | 0.37 | 1 | 3 | 5 | Chuanxiong |
| DSS225 | senkyunolide-F | 206.26 | 1.78 | 0.61 | 1 | 3 | 2 | Chuanxiong |
| DSS226 | senkyunolide-J | 226.3 | 1.15 | 0.02 | 2 | 4 | 3 | Chuanxiong |
| DSS227 | senkyunolide-L | 242.72 | 2.12 | 0.63 | 1 | 3 | 2 | Chuanxiong |
| DSS228 | 1-Acetyl-β-carboline | 210.25 | 2.14 | 1.18 | 1 | 2 | 1 | Chuanxiong |
| DSS229 | senkyunone | 326.52 | 4.92 | 1.15 | 0 | 2 | 8 | Chuanxiong |
| DSS230 | sinapic acid | 224.23 | 1.31 | 0.48 | 2 | 5 | 4 | Chuanxiong |
| DSS231 | 1H-Cycloprop(e)azulen-7-ol, decahydro-1,1,7-trimethyl-4-methylene-,(1aR-(1aα,4aα,7β,7aβ,7bα))- | 220.39 | 3.30 | 1.37 | 1 | 1 | 0 | Chuanxiong |
| DSS232 | trans-2-Nonen-1-ol | 142.27 | 2.64 | 1.17 | 1 | 1 | 6 | Chuanxiong |
| DSS233 | TML | 59.13 | 0.57 | 1.78 | 0 | 1 | 0 | Chuanxiong |
| DSS234 | wallichilide | 412.57 | 4.16 | 0.82 | 0 | 5 | 8 | Chuanxiong |
| DSS235 | 1-Octanol,2,7-dimethyl- | 158.32 | 3.07 | 1.25 | 1 | 1 | 6 | Chuanxiong |
| DSS236 | 1-terpineol | 154.28 | 2.31 | 1.24 | 1 | 1 | 1 | Chuanxiong |
| DSS237 | 1-β-ethylacrylate-7-aldehyde-β-carboline | 294.33 | 2.26 | 0.45 | 1 | 4 | 5 | Chuanxiong |
| DSS238 | 2-Propionylfuran | 124.15 | 1.24 | 1.21 | 0 | 2 | 2 | Chuanxiong |
| DSS239 | WLN: 2VR | 134.19 | 2.12 | 1.45 | 0 | 1 | 2 | Chuanxiong |
| DSS240 | 2,2,3-Trimethylcyclopent-3-ene-1-carboxaldehyde | 138.23 | 1.93 | 1.31 | 0 | 1 | 1 | Chuanxiong |
| DSS241 | methyl 2-pentanoylbenzoate | 220.29 | 2.54 | 0.91 | 0 | 3 | 6 | Chuanxiong |
| DSS242 | ISOHEPTANE | 100.23 | 3.25 | 1.81 | 0 | 0 | 3 | Chuanxiong |
| DSS243 | WLN: T5OJ BVO1 | 126.12 | 0.96 | 1.12 | 0 | 3 | 2 | Chuanxiong |
| DSS244 | 2-Methyl-1-phenylpropene | 132.22 | 3.41 | 1.89 | 0 | 0 | 1 | Chuanxiong |
| DSS245 | 2-methyl-5-(1-methylene)-1,3-cyclohexadiene | 106.18 | 2.13 | 1.83 | 0 | 0 | 0 | Chuanxiong |
| DSS246 | (4S,6S)-cis-Carveol | 152.26 | 2.47 | 1.22 | 1 | 1 | 1 | Chuanxiong |
| DSS247 | 2-Methylbenzoxazol | 133.16 | 1.97 | 1.31 | 0 | 2 | 0 | Chuanxiong |
| DSS248 | (5S,6R)-5,6-dimethyltetrahydropyran-2-one | 128.19 | 1.45 | 1.15 | 0 | 2 | 0 | Chuanxiong |
| DSS249 | 3(S)-3-Butyl-4,5-dihydrophthalide | 194.3 | 2.84 | 1.28 | 0 | 2 | 3 | Chuanxiong |
| DSS250 | 3,4-epoxy-2,2,7,7-tetramethyl-octane | 184.36 | 3.51 | 1.56 | 0 | 1 | 4 | Chuanxiong |
| DSS251 | 3-cyclohexen-1-ol | 98.16 | 1.24 | 1.14 | 1 | 1 | 0 | Chuanxiong |
| DSS252 | Methyl 3-furoate | 126.12 | 1.05 | 1.03 | 0 | 3 | 2 | Chuanxiong |
| DSS253 | trans-Piperitol | 154.28 | 2.30 | 1.19 | 1 | 1 | 1 | Chuanxiong |
| DSS254 | 4,7-Dihydroxy-3-butylphthalide | 222.26 | 2.23 | 0.69 | 2 | 4 | 3 | Chuanxiong |
| DSS255 | 4-iodoindoline | 245.07 | 2.22 | 1.79 | 1 | 1 | 0 | Chuanxiong |
| DSS256 | 4-hydroxy-3-butylphthalide | 206.26 | 2.28 | 0.90 | 1 | 3 | 3 | Chuanxiong |
| DSS257 | (-)-spathulenol | 220.39 | 3.30 | 1.45 | 1 | 1 | 0 | Chuanxiong |
| DSS258 | 5-Propyl-2-thiouracil | 170.26 | 1.07 | 0.93 | 2 | 3 | 2 | Chuanxiong |
| DSS259 | 7-oxabicyclo-2.2.1-heptane,1-methyl-4-[1-methylethyl]- | 154.28 | 2.71 | 1.53 | 0 | 1 | 1 | Chuanxiong |
| DSS260 | Aromadendrene oxide 2 | 220.39 | 3.53 | 1.56 | 0 | 1 | 0 | Chuanxiong |
| DSS261 | Amylbenzene | 148.27 | 3.90 | 1.88 | 0 | 0 | 4 | Chuanxiong |
| DSS262 | Dimethyl D-malate | 162.16 | -0.14 | 0.11 | 1 | 5 | 5 | Chuanxiong |
| DSS263 | dl-3n-butylphthalide | 190.26 | 2.71 | 1.30 | 0 | 2 | 3 | Chuanxiong |
| DSS264 | Cedrene | 204.39 | 4.57 | 1.82 | 0 | 0 | 0 | Chuanxiong |
| DSS265 | carotol | 222.41 | 3.58 | 1.46 | 1 | 1 | 1 | Chuanxiong |
| DSS266 | Coniferylfcrulate | 356.4 | 3.15 | 0.67 | 2 | 6 | 8 | Chuanxiong |
| DSS267 | Cerulignol | 166.24 | 2.36 | 1.42 | 1 | 2 | 3 | Chuanxiong |
| DSS268 | Decahydro-1,6-bis(methylene)-4-(1-methylethyl)-naphthalene | 204.39 | 4.24 | 1.84 | 0 | 0 | 1 | Chuanxiong |
| DSS269 | Hexaphenone | 176.28 | 3.22 | 1.49 | 0 | 1 | 5 | Chuanxiong |
| DSS270 | (E,E)-1,3,5-Undecatriene | 150.29 | 3.95 | 1.84 | 0 | 0 | 6 | Chuanxiong |
| DSS271 | Heptan | 100.23 | 3.54 | 1.77 | 0 | 0 | 4 | Chuanxiong |
| DSS272 | L-valyl-L-valinc-achydride | 214.35 | 0.82 | -0.15 | 4 | 4 | 6 | Chuanxiong |
| DSS273 | Levistolide-A | 380.52 | 4.54 | 0.96 | 0 | 4 | 4 | Chuanxiong |
| DSS274 | tetramethylpyrazine | 136.22 | 1.38 | 1.19 | 0 | 2 | 0 | Chuanxiong |
| DSS275 | (2-amylphenyl)methanol | 178.3 | 3.02 | 1.26 | 1 | 1 | 5 | Chuanxiong |
| DSS276 | 1(3H)-Isobenzofuranone, 3-butyl-3a,4,5,6-tetrahydro-, cis-(-)- | 194.3 | 2.85 | 1.25 | 0 | 2 | 3 | Chuanxiong |
| DSS277 | Senkyunolide A | 192.28 | 2.67 | 1.30 | 0 | 2 | 3 | Chuanxiong |
| DSS278 | Senkyunolide G | 208.28 | 2.01 | 0.63 | 1 | 3 | 3 | Chuanxiong |
| DSS279 | (3Z,6S,7S)-3-butylidene-6,7-dihydroxy-4,5,6,7-tetrahydroisobenzofuran-1-one | 224.28 | 1.07 | 0.00 | 2 | 4 | 2 | Chuanxiong |
| DSS280 | Germacrene D | 204.39 | 4.37 | 1.83 | 0 | 0 | 1 | Chuanxiong |
| DSS281 | Cerevisterol | 430.74 | 5.00 | 0.28 | 3 | 3 | 4 | Fuling |
| DSS282 | Dimethyl L-malate | 162.16 | -0.22 | -0.05 | 1 | 5 | 5 | Fuling |
| DSS283 | Trimethyl citrate | 234.23 | -0.14 | -0.06 | 1 | 7 | 8 | Fuling |
| DSS284 | 2-lauroleic acid | 198.34 | 3.66 | 1.03 | 1 | 2 | 9 | Fuling |
| DSS285 | caprylic acid | 144.24 | 2.35 | 0.90 | 1 | 2 | 6 | Fuling |
| DSS286 | (+/-)-Isoborneol | 154.28 | 2.41 | 1.27 | 1 | 1 | 0 | Baizhu |
| DSS287 | 12-senecioyl-2E,8E,10E-atractylentriol | 312.39 | 2.79 | 0.01 | 0 | 4 | 8 | Baizhu |
| DSS288 | α-Longipinene | 204.39 | 4.49 | 1.83 | 0 | 0 | 0 | Baizhu |
| DSS289 | β-Humulene | 204.39 | 4.42 | 1.82 | 0 | 0 | 0 | Baizhu |
| DSS290 | (1R)-2-methyl-1-phenylprop-2-en-1-ol | 148.22 | 2.24 | 1.27 | 1 | 1 | 2 | Baizhu |
| DSS291 | (3S)-3-[(1R)-1,5-dimethylhex-4-enyl]-6-methylenecyclohexene | 204.39 | 4.61 | 1.88 | 0 | 0 | 4 | Baizhu |
| DSS292 | β-Eudesmol | 222.41 | 3.60 | 1.32 | 1 | 1 | 1 | Baizhu |
| DSS293 | β-caryophyllene | 204.39 | 4.26 | 1.83 | 0 | 0 | 0 | Baizhu |
| DSS294 | γ-elemene | 204.39 | 4.54 | 1.87 | 0 | 0 | 2 | Baizhu |
| DSS295 | Akridin | 179.23 | 3.02 | 1.63 | 0 | 1 | 0 | Baizhu |
| DSS296 | (1S,2R,4R)-Neoiso-dihydrocarveol | 154.28 | 2.57 | 1.38 | 1 | 1 | 1 | Baizhu |
| DSS297 | Phenylalanine | 165.21 | -0.23 | 0.36 | 3 | 3 | 3 | Baizhu |
| DSS298 | LPG | 89.11 | -1.57 | -0.34 | 3 | 3 | 1 | Baizhu |
| DSS299 | Atractylenolide I | 230.33 | 3.15 | 1.30 | 0 | 2 | 0 | Baizhu |
| DSS300 | Atractylenolide II | 232.35 | 3.09 | 1.30 | 0 | 2 | 0 | Baizhu |
| DSS301 | Atractylenolide III | 248.35 | 2.53 | 0.75 | 1 | 3 | 0 | Baizhu |
| DSS302 | atractylone | 216.35 | 3.61 | 1.76 | 0 | 1 | 0 | Baizhu |
| DSS303 | juniper camphor | 222.41 | 3.64 | 1.44 | 1 | 1 | 0 | Baizhu |
| DSS304 | (5E,9Z)-3,6,10-trimethyl-4,7,8,11-tetrahydrocyclodeca[b]furan | 216.35 | 3.61 | 1.77 | 0 | 1 | 0 | Baizhu |
| DSS305 | 3β-acetoxyatractylone | 274.39 | 3.10 | 1.13 | 0 | 3 | 2 | Baizhu |
| DSS306 | DTY | 181.21 | -0.69 | -0.10 | 4 | 4 | 3 | Baizhu |
| DSS307 | DIBP | 278.38 | 3.54 | 0.85 | 0 | 4 | 8 | Baizhu |
| DSS308 | 2-[(2R,5S,6S)-6,10-dimethylspiro[4.5]dec-9-en-2-yl]propan-2-ol | 222.41 | 3.62 | 1.34 | 1 | 1 | 1 | Baizhu |
| DSS309 | selina-4(14),7(11)-dien-8-one | 218.37 | 3.51 | 1.42 | 0 | 1 | 0 | Baizhu |
| DSS310 | Prolinum | 115.15 | -1.20 | 0.22 | 2 | 3 | 1 | Baizhu |
| DSS311 | alloaromadedrene | 204.39 | 4.46 | 1.83 | 0 | 0 | 0 | Baizhu |
| DSS312 | L-Valin | 117.17 | -0.85 | 0.04 | 3 | 3 | 2 | Baizhu |
| DSS313 | L-Ile | 131.2 | -0.45 | 0.06 | 3 | 3 | 3 | Baizhu |
| DSS314 | Ethyl pivaloylacetate | 172.25 | 1.66 | 0.82 | 0 | 3 | 5 | Baizhu |
| DSS315 | 8β-ethoxy atractylenolide Ⅲ | 276.41 | 3.24 | 1.08 | 0 | 3 | 2 | Baizhu |
| DSS316 | choline | 104.2 | -0.65 | 0.86 | 1 | 1 | 2 | Zexie |
| DSS317 | emodin | 270.25 | 1.70 | 0.22 | 3 | 5 | 0 | Zexie |
| DSS318 | HMF | 126.12 | -0.08 | 0.05 | 1 | 3 | 2 | Zexie |
| DSS319 | 1h-indole-3-carboxylic,acid | 161.17 | 1.49 | 0.79 | 2 | 2 | 1 | Zexie |
| DSS320 | (1R,3aS,8aR)-7-isopropyl-1-methyl-4-methylene-2,3,3a,5,6,8a-hexahydroazulen-1-ol | 220.39 | 3.26 | 1.24 | 1 | 1 | 1 | Zexie |
| DSS321 | (1R,3aR,4S,7R,8S,8aS)-7-isopropyl-1,4-dimethyl-3,3a,5,6,8,8a-hexahydro-2H-azulene-1,4,7,8-tetrol | 272.43 | 1.33 | -0.03 | 4 | 4 | 1 | Zexie |
| DSS322 | (1R,3aS,4S,8aR)-7-(2-hydroxypropan-2-yl)-1,4-dimethyl-2,3,3a,5,6,8a-hexahydroazulene-1,4-diol | 254.41 | 1.75 | -0.13 | 3 | 3 | 1 | Zexie |
| DSS323 | (1S,3aR,4R,8aS)-7-isopropyl-1,4-dimethyl-2,3,3a,5,6,8a-hexahydroazulene-1,4-diol | 238.41 | 2.64 | 0.62 | 2 | 2 | 1 | Zexie |
| DSS324 | Alisol B | 472.78 | 4.85 | 0.04 | 2 | 4 | 4 | Zexie |
| DSS325 | alisol,b,23-acetate | 446.74 | 4.51 | -0.06 | 2 | 4 | 4 | Zexie |
| DSS326 | lismol | 220.39 | 3.25 | 1.27 | 1 | 1 | 1 | Zexie |
| DSS327 | rel-(1R,3aS,4S,7S,8R,8aS)-7-isopropyl-1,4-dimethyldecahydro-4,7-epoxyazulene-1,8-diol | 254.41 | 1.95 | 0.53 | 2 | 3 | 1 | Zexie |
| DSS328 | orientalol,f | 236.39 | 2.81 | 1.15 | 1 | 2 | 1 | Zexie |
| DSS329 | Sulfoorientalol A | 302.48 | 2.39 | 0.27 | 2 | 4 | 2 | Zexie |
| DSS330 | Sulfoorientalol B | 318.48 | 1.80 | -0.24 | 3 | 5 | 3 | Zexie |
| DSS331 | Sulfoorientalol C | 300.46 | 2.39 | 0.06 | 2 | 4 | 2 | Zexie |
| DSS332 | Sulfoorientalol D | 318.48 | 1.87 | 0.38 | 2 | 5 | 2 | Zexie |
| DSS333 | alisol A | 490.8 | 3.99 | -0.20 | 4 | 5 | 5 | Zexie |
| DSS334 | alisol B | 444.72 | 4.19 | 0.07 | 2 | 4 | 4 | Zexie |
| DSS335 | alisol C | 486.76 | 4.00 | -0.34 | 2 | 5 | 4 | Zexie |
| DSS336 | NCA | 122.14 | 0.02 | 0.44 | 2 | 3 | 1 | Zexie |

**Table S3.** The detail information of network core intervention motifs (CIM) in DSS.

| CIM | Gene list | P value | FDR value |
| --- | --- | --- | --- |
| 1 | ABCC4, ACLY, ADA, ADCY5, ADORA1, ADORA2A, ADORA2B, AHCY, AHCYL1, AHCYL2, AKR1B1, AKT1, AKT2, AKT3, ALOX5, AR, AZU1, BCL2, BCL2A1, BCL2L1, BRSK2, CBR1, CDC25A, CDC25B, CDK1, CDK2&CCNE1, CDK4, CNR1, CRYZ, CSNK2A1, CSNK2A2, CSNK2A3, CSNK2B, CYP19A1, DBH, DNMT3B, DRD2, DRD3, DRD4, DUSP3, DYRK1A, ELANE, ELAVL1, ELAVL2, ELAVL3, ELAVL4, ESR1, ESR2, F2, FGB, FLT1, FLT4, GDA, GRM2, GSK3A, GSK3B, GSR, HMGCR, HPRT1, HRH3, HSD11B1, HSD11B1L, HSD17B12, HSD17B3, HSP90AA1, HSP90AB1, HSP90B1, HTR2A, IDO1, IMPDH1, IMPDH2, JUN, JUNB, JUND, KCNB1, KDR, MAP2, MAPK10, MAPK11, MAPK14, MAPK8, MAPK9, MAPT, MBNL1, MBNL2, MBNL3, MCL1, MGMT, MKNK1, MPEG1, MPG, MTAP, NAT1, NR0B1, NR1H4, NR3C1, NR3C2, NSD2, NUDT1, OPRD1, OPRK1, OPRL1, OPRM1, PDE2A, PIM1, PLCG1, PNP, POLA1, POLH, POLK, POLL, POLM, PON1, PPM1A, PPM1B, PPP1CA, PPP1CB, PPP1CC, PPP2R1A, PPP2R1B, PPP5C, PRKDC, PRTFDC1, PRTN3, PTAFR, PTGS1, PTP4A1, PTP4A2, PTP4A3, PTPN1, PTPN2, SIRT5, SLC37A4, SLC47A2, SMARCA2, SRD5A2, SRPK2, STAT1, STAT2, STAT3, STAT4, TCF4, TDP1, TERT, TP53 | 0.0001 | 0.00178 |
| 2 | ABCB1, ABCB11, ABCB4, ABCB5, AR, ELANE, KAT2A, OPRD1, OPRK1, OPRL1, OPRM1, PRKCA, PRKCB, PRKCG, PRKCQ, RASGRP3, SLC6A2, SLC6A3, SMAD3 | 0.00178 | 0.0001 |
| 3 | ABAT, ABCB1, ABCB11, ABCB4, ABCB5, ABCC1, ABCC9, ABCG2, ABHD6, ACE, ACE2, ACER2, ACHE, ACLY, ACP1, ACPP, ACR, ADA, ADAM12, ADAM17, ADCYAP1R1, ADH1A, ADH1B, ADH1C, ADH4, ADH5, ADH6, ADH7, ADORA1, ADORA2A, ADORA2B, ADORA3, ADRA1A, ADRA1B, ADRA1D, ADRA2A, ADRA2B, ADRA2C, ADRB1, ADRM1, AGER, AGTR1, AHCY, AHR, AKR1A1, AKR1B1, AKR1B10, AKR1B15, AKR1C1, AKR1C2, AKR1C3, AKR1C4, AKR1D1, AKR1E2, AKT1, AKT1S1, AKT2, AKT3, ALB, ALDH1A1, ALDH2, ALDH3A1, ALDH5A1, ALOX12, ALOX12B, ALOX15, ALOX15B, ALOX5, ALOXE3, ALPG, ALPI, ALPL, ALPP, ALPPL2, AMPD1, AMPD2, AMY1A; AMY1B; AMY1C, ANPEP, AOC2, AOC3, APAF1, APEX1, APLNR, APLP2, APOBEC3A, APOBEC3G, APP, AR, ARG1, ASAH1, ATF1, ATG4B, ATIC, ATR, AVPR1A, AZU1, BACE1, BAZ2A, BAZ2B, BBOX1, BCHE, BCL2, BCL2A1, BCL2L1, BDKRB1, BHMT, BMP1, BRAF, BRPF1, BRS3, BRSK1, BST1, C1R, C3AR1, CA1, CA12, CA13, CA14, CA2, CA3, CA4, CA5A, CA5B, CA6, CA7, CA9, CACNA1A, CACNA1D, CACNA1G, CACNA1I, CACNA2D1, CACNB1, CALM1, CAMK2A, CAMK2B, CAMK2D, CAMK2G, CAMKK2, CAPN1, CAPN2, CAPNS1, CARM1, CASP1, CASR, CAT, CBR1, CBX7, CCBL1, CCNC, CCND3, CCR2, CCR4, CCR6, CCR9, CDC25A, CDC25B, CDC25C, CDC7, CDK1, CDK2, CDK3, CDK4, CDK6, CDK8, CELA1, CES1, CES2, CES3, CES5A, CFTR, CHEK1, CHEK2, CHIT1, CHRM1, CHRM2, CHRM3, CHRM4, CHRM5, CHRNA1, CHRNA2, CHRNA3, CHRNA4, CHRNA5, CHRNA7, CHRNB1, CHRNB2, CHRNB4, CHRND, CHRNG, CISD1, CLC, CLK1, CMA1, CNOT7, CNR1, CNR2, COL4A3BP, COMT, COQ8B, CPA1, CPA3, CPB1, CPB2, CPN1, CPT1A, CPT1B, CPT2, CRABP1, CRABP2, CREB1, CREBBP, CRYZ, CSF1R, CSK, CSNK1G2, CSNK1G3, CSNK2A1, CSNK2A2, CSNK2A3, CSNK2B, CTBP2, CTDSP1, CTRB1, CTRC, CTRL, CTSA, CTSB, CTSC, CTSG, CTSH, CTSK, CTSL, CTSS, CXCL8, CXCR5, CYP11B1, CYP11B2, CYP17A1, CYP19A1, CYP1A1, CYP1A2, CYP1B1, CYP24A1, CYP26A1, CYP26B1, CYP2A6, CYP2B6, CYP2C18, CYP2C19, CYP2C8, CYP2C9, CYP2D6, CYP2E1, CYP2J2, CYP3A4, CYP4F2, CYP4F22, CYP4F3, CYP51A1, DAGLA, DAO, DAPK1, DAPK2, DAPK3, DBF4, DBH, DBI, DCXR, DDO, DDX3X, DHCR7, DHFR, DHH, DHODH, DLG4, DNM1, DNM2, DNMT3A, DNMT3L, DNPEP, DPP4, DPP7, DPP8, DPP9, DRD1, DRD2, DRD3, DRD4, DUSP22, DUSP3, DUSP6, DUSP7, DYRK1A, DYRK1B, DYRK2, DYRK3, DYRK4, EBP, EBPL, ECE1, EDNRA, EDNRB, EGFR, EGLN1, EGLN2, EGLN3, EIF2AK3, EIF4E, EIF4H, ELANE, ELAVL1, ENPEP, ENPP2, EP300, EPHB2, EPHX1, EPHX2, ERAP1, ERAP2, ERBB2, ERBB3, ERBB4, ERCC1, ERCC4, ERCC5, ERN1, ESR1, ESR2, ESRRA, ESRRB, ESRRG, EWS-Fli1, F10, F11, F12, F2, F3, FAAH, FABP1, FABP12, FABP2, FABP3, FABP4, FABP5, FABP7, FABP9, FADS1, FAP, FCER2, FDFT1, FDPS, FEN1, FFAR1, FFAR4, FGB, FGR, FKBP1A, FLT3, FNTA, FNTA&FNTB, FNTB, FOLH1, FOS, FPR1, FTO, FUCA1, FUCA2, FUT4, FUT7, FYN, G6PC, G6PD, GAA, GABBR1, GABBR2, GABBR2&GABBR1, GABRA1, GABRA2, GABRA4, GABRA6, GABRB1, GABRB2, GABRB2&GABRG2&GABRA1, GABRG2, GABRQ, GABRR1, GABRR2, GALR2, GBA, GBA2, GCGR, GCLC, GFER, GFPT1, GGH, GGPS1, GHSR, GLB1, GLI1, GLO1, GLRA4, GLTP, GNAI1, GNAI3, GNAO1, GNB1, GNG2, GNPAT, GPR139, GPR174, GPR183, GPR34, GPR35, GPR84, GRB2, GRIA1, GRIA2, GRIA3, GRIA4, GRIK1, GRIK2, GRIK3, GRIK5, GRIN1, GRIN1&GRIN2B, GRIN2B, GRK6, GRM1, GRM3, GRM4, GRM5, GRM6, GRM7, GRM8, GSK3A, GSK3B, GSR, GSTA1, GSTK1, GSTM1, GSTM2, GSTP1, GUSB, HAAO, HAO1, HAO2, HCAR1, HCAR2, HCAR3, HCN4, HDAC1, HDAC10, HDAC11, HDAC2, HDAC3, HDAC4, HDAC5, HDAC6, HDAC7, HDAC8, HDAC9, HGFAC, HIF1AN, HKDC1, HLA-A, HLA-DRB1, HMGCR, HMOX1, HNF4A, HPGDS, HPSE, HRH1, HRH2, HSD11B1, HSD11B1L, HSD17B1, HSD17B10, HSD17B12, HSD17B14, HSD17B2, HSD17B3, HSP90AA1, HSP90AB1, HSP90B1, HSPA1A, HSPB1, HSPD1, HTR1A, HTR1B, HTR1D, HTR1E, HTR1F, HTR2A, HTR2B, HTR2C, HTR4, HTR6, HTR7, I3L2F9, ICMT, IDE, IDO1, IGF2R, IGFBP1, IGFBP2, IGFBP3, IGFBP4, IGFBP5, IGFBP6, IGHG4, IHH, IKBKB, IKBKG, IL1B, IL2, IL6ST, IMPDH1, IMPDH2, ITGA2B, ITGA4, ITGB1, JUN, KAT2A, KAT2B, KCNA1, KCNA2, KCNA3, KCNA4, KCNA5, KCNB1, KCND3, KCNH3, KCNJ11, KCNK2, KCNK3, KCNK9, KCNMA1, KCNN1, KCNN2, KCNN3, KCNN4, KDM1A, KDM2A, KDM2B, KDM3A, KDM4A, KDM4C, KDM4D, KDM4E, KDM5A, KDM5B, KDM5C, KDM6A, KDM6B, KDM7A, KEAP1, KIF11, KIF20A, KIF20B, KLF5, KLK1, KLK14, KLK2, KLK4, KLK5, KLK7, KLK8, KLKB1, KMO, KYNU, L3MBTL3, LAP3, LCK, LCN2, LDHA, LDHB, LDLR, LIG1, LIPE, LIPF, LNPEP, LOXL2, LPAR1, LPAR2, LPAR3, LPAR4, LPAR5, LPAR6, LRRK2, LTA4H, LTB4R, LTC4S, LYPLA1, LYPLA2, MALT1, MAN2B1, MAOA, MAOB, MAP2K3, MAP3K7, MAP4K4, MAPK8, MAPK9, MAPT, MARK4, MARS, MAX, MB, MBD2, MBNL1, MBNL2, MBNL3, MC1R, MC3R, MC4R, MC5R, MCL1, MDH1, MDH2, MDM4, MECP2, MET, METAP1, METAP2, MGAM, MGLL, MIF, MITF, MLYCD, MME, MMEL1, MMP1, MMP10, MMP12, MMP13, MMP14, MMP15, MMP16, MMP2, MMP26, MMP3, MMP7, MMP8, MMP9, MPEG1, MPG, MPI, MPL, MPO, MT-ND1, MT-ND2, MT-ND3, MT-ND4, MT-ND4L, MT-ND5, MT-ND6, MTNR1A, MTNR1B, MYC, MYLK, NAAA, NAALAD2, NAALADL1, NAMPT, NCEH1, NCOA1, NCOA2, NCOR1, NCOR2, NDUFA1, NDUFA10, NDUFA11, NDUFA12, NDUFA13, NDUFA2, NDUFA3, NDUFA4, NDUFA4L2, NDUFA5, NDUFA6, NDUFA7, NDUFA8, NDUFA9, NDUFAB1, NDUFAF1, NDUFAF2, NDUFAF3, NDUFAF4, NDUFB1, NDUFB10, NDUFB11, NDUFB2, NDUFB3, NDUFB4, NDUFB5, NDUFB6, NDUFB7, NDUFB8, NDUFB9, NDUFC1, NDUFC2, NDUFS1, NDUFS2, NDUFS3, NDUFS4, NDUFS5, NDUFS6, NDUFS7, NDUFS8, NDUFV1, NDUFV2, NDUFV3, NEK6, NEU3, NFE2L2, NFKB1, NGF, NISCH, NLRP1, NLRP3, NMBR, NMUR1, NMUR2, NOD1, NOD2, NOS1, NOS2, NOS3, NOX1, NOX4, NPBWR1, NPFFR1, NPFFR2, NPPA, NQO1, NQO2, NR0B1, NR0B2, NR1H2, NR1H4, NR4A1, NR4A2, NR5A1, NR5A2, NSD2, NUAK1, ODC1, OPRD1, OPRK1, OPRL1, OPRM1, OTC, OXER1, P2RX1, P2RX4, P2RY10, P2RY12, P4HA1, P4HB, P4HTM, PABPC1, PADI1, PADI2, PADI3, PADI4, PAFAH1B2, PAH, PAM, PAOX, PARP1, PARP10, PARP14, PARP15, PARP3, PAX8, PBK, PBRM1, PCNA, PDCD4, PDE3A, PDE3B, PDE4B, PDE4C, PDE4D, PDF, PDGFRA, PDK1, PDK3, PDK4, PDYN, PEPD, PGA5, PGD, PGF, PGR, PHF8, PHGDH, PHKG2, PHLPP2, PHOSPHO1, PIK3C2A, PIK3C2G, PIM1, PIM2, PIM3, PIN1, PIN4, PKN1, PLA2G10, PLA2G1B, PLA2G2A, PLA2G2C, PLA2G2D, PLA2G2E, PLA2G2F, PLA2G4A, PLA2G4B, PLA2G4C, PLA2G5, PLAA, PLAT, PLAU, PLAUR, PLCG1, PLCG2, PLEC, PMM2, PMP2, POLA1, POLB, POLH, POLI, POLK, POLL, POLM, PON1, PPARA, PPARD, PPARG, PPIA, PPID, PPM1A, PPM1B, PPP1CC, PRKAA1, PRKAA2, PRKAB1, PRKAB2, PRKACA, PRKAG1, PRKCA, PRKCB, PRKCD, PRKCE, PRKCG, PRKCH, PRKCQ, PRMT6, PRNP, PRSS1, PRSS2, PRSS3, PRTN3, PSMA1, PSMA2, PSMA3, PSMA4, PSMA5, PSMA6, PSMA7, PSMA8, PSMB1, PSMB11, PSMB3, PSMB4, PSMB5, PSMB6, PSMB7, PSMC1, PSMC2, PSMC3, PSMC4, PSMC5, PSMC6, PSMD1, PSMD11, PSMD12, PSMD13, PSMD14, PSMD2, PSMD3, PSMD4, PSMD6, PSMD7, PSMD8, PTAFR, PTGDR2, PTGER1, PTGER2, PTGER3, PTGER4, PTGES, PTGFR, PTGIR, PTGR1, PTGS1, PTGS2, PTK2, PTPN1, PTPN11, PTPN12, PTPN13, PTPN2, PTPN6, PTPN7, PTPRA, PTPRC, PTPRF, PTPRG, PTPRS, PTPsigma, PYGL, QDPR, QPCTL, QTRT1, RABGGTB, RAD51, RAD52, RAF1, RAPGEF4, RARA, RARB, RARG, RASGRP1, RASGRP3, RBBP9, RBP4, RCE1, RELA, REN, RET, RHO, RHOA, RIPK1, RNASE1, RNASEH1, RNPEP, ROCK1, ROCK2, RORA, RORB, RORC, RPA1, RPS6KA3, RPS6KA4, RPS6KB2, RRM1, RUVBL1, RXRA, RXRB, RXRG, RYR2, S1PR1, S1PR2, S1PR3, S1PR4, S1PR5, SAE1, SCARB1, SCN10A, SCN2A, SCN3A, SCN4A, SCN5A, SCN8A, SCNN1A, SELE, SELL, SELP, SEM1, SENP1, SENP6, SENP7, SENP8, SERPINE1, SGK1, SGK2, SHBG, SHH, SI, SIGMAR1, SIRT1, SIRT2, SIRT3, SIRT5, SLC13A5, SLC15A1, SLC16A1, SLC16A10, SLC16A2, SLC16A3, SLC18A2, SLC18A3, SLC1A1, SLC1A2, SLC1A3, SLC1A5, SLC22A1, SLC22A10, SLC22A11, SLC22A12, SLC22A2, SLC22A24, SLC22A25, SLC22A3, SLC22A5, SLC22A6, SLC22A7, SLC22A8, SLC22A9, SLC25A20, SLC27A4, SLC2A1, SLC2A4, SLC37A4, SLC5A7, SLC6A1, SLC6A11, SLC6A14, SLC6A2, SLC6A3, SLC6A4, SLC6A5, SLC6A7, SLC6A9, SLC7A11, SLC7A5, SLC7A8, SLC9A1, SLC9A2, SLCO2A1, SLCO2B1, SLCO4C1, SMAD3, SMN1; SMN2, SMPD2, SNCA, SOAT1, SOAT2, SORT1, SPHK1, SPHK2, SPR, SQLE, SRC, SRD5A2, SRPK1, SSTR1, SSTR3, SSTR4, SSTR5, ST3GAL1, ST3GAL3, ST6GAL1, STAT1, STK11, STK17A, STK17B, STK38L, STS, SULT1A1, SULT1A2, SULT1A4, SULT1B1, SULT1C2, SULT1C3, SULT1C4, SULT1E1, SUV39H1, TAAR1, TAAR2, TAAR5, TAB1, TACR1, TACR2, TACR3, TAOK1, TAOK3, TAS1R1, TAS1R2, TAS1R3, TAS2R14, TAS2R31, TAT, TBXA2R, TBXAS1, TCF4, TDO2, TDP1, TDP2, TERT, TGM1, TGM2, TGM3, TH, THRA, THRB, TLR2, TLR7, TLR8, TLR9, TMIGD3, TMPRSS6, TNFRSF1A, TNKS, TNKS2, TOP1, TOP2A, TPH1, TPH2, TPMT, TRPA1, TRPC3, TRPC6, TRPM2, TRPM5, TRPM8, TRPV1, TSPO, TSSK2, TTR, TUBB1, TUBB3, TUBB8, TXK, TXNRD1, TYMP, TYMS, TYR, UBA2, UBE2I, UBLCP1, UCHL1, UGT1A1, UGT2A1, UGT2A3, UGT2B10, UGT2B11, UGT2B15, UGT2B17, UGT2B28, UGT2B4, UGT2B7, UNG, UQCRB, USP1, USP4, USP5, USP7, VEGFA, VHL, WDR48, XBP1, XDH, XIAP, XPNPEP1, XPNPEP2, XPO1, YARS, YES1, YWHAG | 0.00445 | 0.0001 |
| 4 | ABCB1, ABCG2, ADCYAP1R1, AKR1B1, AKR1B10, AKR1C3, AKR1C4, ALOX12, ALOX15, ALOX15B, ALOX5, ALOXE3, ALPI, AOC3, APP, BACE1, BCHE, BRAF, CA1, CA12, CA13, CA14, CA2, CA3, CA4, CA5A, CA5B, CA6, CA7, CA9, CALM1, CAMK2A, CCND3, CISD1, CSNK1G3, CYP1A1, CYP1B1, DHCR7, DNM1, DRD1, DRD5, DUSP3, DYRK1A, DYRK4, EBP, EP300, ERN1, F3, FOS, GFER, GLO1, GPR183, GRIK1, HMGCR, HSPD1, IGF1R, IGF2R, IGFBP5, IKBKG, INSR, JUN, KDM4E, KLF5, LYPLA1, LYPLA2, MAOA, MAOB, MAPT, MBNL1, MBNL2, MBNL3, MCL1, MET, MMP1, MMP2, MMP9, MPO, MT-ND1, MT-ND2, MT-ND3, MT-ND4, MT-ND4L, MT-ND5, MT-ND6, MTNR1A, MTNR1B, NDUFA1, NDUFA10, NDUFA11, NDUFA12, NDUFA13, NDUFA2, NDUFA3, NDUFA4, NDUFA4L2, NDUFA5, NDUFA6, NDUFA7, NDUFA8, NDUFA9, NDUFAB1, NDUFAF1, NDUFAF2, NDUFAF3, NDUFAF4, NDUFB1, NDUFB10, NDUFB11, NDUFB2, NDUFB3, NDUFB4, NDUFB5, NDUFB6, NDUFB7, NDUFB8, NDUFB9, NDUFC1, NDUFC2, NDUFS1, NDUFS2, NDUFS3, NDUFS4, NDUFS5, NDUFS6, NDUFS7, NDUFS8, NDUFV1, NDUFV2, NDUFV3, NFE2L2, NFKB1, NOD2, NQO2, NR0B2, NR1H4, ODC1, PTGER2, PTGES, PTPN1, PTPN6, PTPN7, QDPR, RAF1, RELA, SHBG, SIGMAR1, SLC22A1, SLC22A3, SPR, STK17B, TAAR1, TAAR5, TDP1, TLR9, TNFRSF1A, TOP2A, TRPM8, TRPV1, TTR, TUBB1, TUBB3 | 0.002967 | 0.0001 |
| 5 | ACHE, AR, BCHE, CA1, CA13, CA2, CA3, CA4, CA5A, CA5B, CA7, CDC25A, CDC25B, CHRM1, CHRM2, CHRM3, CHRM4, CHRM5, CHRNA2, CXCR3, CYP17A1, CYP19A1, CYP24A1, CYP3A4, CYP3A5, CYP51A1, DHH, ESR1, ESR2, FAAH, FUT7, G6PD, GRIN1&GRIN2B, HMGCR, HSD11B1, HSD11B1L, HSD11B2, HSD17B12, HSD17B3, IHH, LCN9, LDLR, LRP8, MAPT, NISCH, NR1H2, NR1H3, OPRK1, PGR, PTGS1, PTGS2, REN, SHH, SLC22A1, SLC22A2, SLC22A3, SLC5A7, SLC6A2, SLC6A3, SRD5A1, TBXAS1, TDP1, TRPA1, UGT2A1, UGT2A3, UGT2B10, UGT2B11, UGT2B15, UGT2B17, UGT2B28, UGT2B4, UGT2B7, VDR, VLDLR | 0.002444 | 0.000165 |
| 6 | ABCC4, ADAM28, ADRA2A, ADRA2B, ADRA2C, AR, CHRM1, CHRM2, CHRM3, CHRM4, CHRM5, CTRC, CTSG, DPP4, DRD2, DRD3, DRD4, ESR1, ESR2, FAP, HTR1A, HTR1B, HTR1D, HTR1E, HTR2A, HTR2B, HTR2C, HTR3A, KCNH2, KCNH6, LDLR, LRP8, MAOA, MAOB, MAPT, MSR1, NISCH, NOS1, NOS2, NOS3, NR1H4, OPRD1, OPRK1, OPRL1, OPRM1, PDE10A, RNPEP, SIGMAR1, SLC6A2, SLC6A3, SLC6A4, SLC6A9, SRD5A2, TAAR1, TAAR2, TDP1, VLDLR | 0.005739 | 0.046438 |

**Table S4.** The self-check list of the ARRIVE guideline.

| **Item** | **Recommendation** | **Check and concrete information** |
| --- | --- | --- |
| Item 6a | The number of experimental and control groups. | 5groups including 4 experimental groups and a control group were designed in the current study. |
| Item 6b | Any steps taken to minimize the effects of subjective bias when allocating animals to treatment (e.g. randomization procedure) and when assessing results (e.g. if done, describe who was blinded and when). | 50 male ICR mice (18–22 g) were randomly divided into five groups when allocating animals to treatment. To avoid the data bias caused by subjective expectations of observers, data was recorded by 2 observers in a double-blind trial, followed by the calculation an average of the 2 groups. |
| Item 8a | Why did the authors only select male mice for the study? | Females are markedly underinvestigated in the behavioral sciences due to the presumption that cyclic hormonal changes across the ovulatory cycle introduce excess variability to measures of interest in comparison to males. Therefore, only male mice were selected for the study. |
| Item 9a | Housing | The mice were housed 5 mice per cage (15.24 cm × 25.40 cm × 12.70 cm), which was prepared with approximately 75 g of wood chip bedding. |
| Item 9c | Welfare assessment | The mice were maintained under a 12 h light-12 h dark cycle and free access to water or food throughout the study. |
| Item 10 | Sample size | The total number of animals used in each experiment was 50, and the number of animals in each experimental group was 10 (n = 10). |
| Item 11 | Allocating animals to experimental groups | 50 male ICR mice were randomly divided into five groups as follows: Control group, FA (25 mg/kg) group, FA (50 mg/kg) group, FA (100 mg/kg) group, and Venlafaxine (50 mg/kg) group. The solutions of drugs were administered to the mice via gastric intubation at a dosage of 0.2 mL/10 g (body weight) once daily between 9:00 a.m. to 10:00 a.m for 14 days. Simultaneously, mice in the control group were orally given vehicle at an equal volume. |
| Item 15 | Numbers analyzed | The number of animals in each group in each analysis was 10. All animals or data were included in the analysis. |
| Item 17 | Adverse events | In the current study, no adverse event was observed in each experimental group. |
| Item 18b | Comment on the study limitations including any potential sources of bias, any limitations of the animal model, and the imprecision associated with the results | In the tail suspension test, some factors may lead to imprecise results, such as the increased noise in the environment, the residual feces and urine in box, etc. Therefore, laboratory environment should be kept quiet, and the feces and urine of mice should be collected after each experiment. In the forced swimming test, the change in height and temperature of water may lead to imprecise results. The height and temperature of water should be maintained during the test period of test. |

**Table S5.** The absorbance value of MTT assay.

|  | Control | Corticosterone | Ferulic acid (2μmol/L) | Ferulic acid (10μmol/L) | Ferulic acid (20μmol/L) |
| --- | --- | --- | --- | --- | --- |
| 1 | 0.6991 | 0.4151 | 0.3736 | 0.4675 | 0.4815 |
| 2 | 0.666 | 0.3645 | 0.4514 | 0.4326 | 0.4434 |
| 3 | 0.6844 | 0.39 | 0.4363 | 0.4566 | 0.4646 |
| 4 | 0.7124 | 0.3939 | 0.4456 | 0.4634 | 0.5021 |

**Table S6.** The immobility time in forced swimming test (FST).

|  | Control | Venlafaxine  (50 mg/kg) | Ferulic acid (25 mg/kg) | Ferulic acid (50 mg/kg) | Ferulic acid (100 mg/kg) |
| --- | --- | --- | --- | --- | --- |
| 1 | 177.83 | 129.71 | 115.4 | 79.28 | 30.92 |
| 2 | 110.71 | 100.98 | 142.01 | 165.97 | 150.24 |
| 3 | 139.44 | 103.61 | 129.16 | 71.23 | 33.78 |
| 4 | 136.40 | 27.32 | 93.40 | 145.68 | 115.87 |
| 5 | 141.62 | 99.09 | 146.81 | 74.72 | 173.11 |
| 6 | 148.97 | 110.75 | 49.97 | 43.54 | 108.18 |
| 7 | 220.42 | 141.24 | 101.74 | 112.55 | 19.41 |
| 8 | 154.22 | 41.86 | 125.47 | 116.69 | 78.94 |
| 9 | 159.06 | 99.47 | 224.10 | 106.00 | 38.41 |
| 10 | 87.90 | 100.78 | 143.84 | 157.14 | 90.00 |

**Table S7.** The immobility time in [tail suspension test](https://www.so.com/link?m=bjm3Kpd5wEnC0yC2qb5uR2MG7j8ePIr7IqiznKZbpu%2FgiKF4Ml23XOizAGJPihvE%2FoTmsBWBiXEDnzL2S11u2kTicE5vVBX2FZGZd9d2jnHvvOctCXHpRRXg%2FuM8Eazgwh6AV22fdbuhJ84FGKRhk%2BIFGyg7DMUyL6GsQwA%3D%3D) (TST).

|  | Control | Venlafaxine (50 mg/kg) | Ferulic acid (25 mg/kg) | Ferulic acid (50 mg/kg) | Ferulic acid (100 mg/kg) |
| --- | --- | --- | --- | --- | --- |
| 1 | 12.50 | 3.40 | 32.60 | 86.60 | 61.41 |
| 2 | 104.21 | 101.47 | 31.66 | 26.31 | 51.50 |
| 3 | 83.56 | 92.50 | 99.51 | 31.44 | 35.01 |
| 4 | 129.27 | 23.48 | 62.87 | 66.64 | 40.94 |
| 5 | 38.30 | 11.41 | 24.41 | 57.58 | 7.53 |
| 6 | 47.70 | 26.10 | 42.37 | 22.75 | 23.80 |
| 7 | 78.83 | 56.03 | 13.59 | 64.26 | 65.18 |
| 8 | 71.22 | 0.81 | 81.41 | 114.07 | 26.03 |
| 9 | 129.64 | 5.32 | 22.68 | 1.28 | 44.23 |
| 10 | 74.83 | 35.60 | 95.47 | 48.93 | 10.20 |
